# Dynein and Dynactin Leverage Their Bivalent Character to Form a High-Affinity Interaction

**DOI:** 10.1371/journal.pone.0059453

**Published:** 2013-04-05

**Authors:** Amanda E. Siglin, Shangjin Sun, Jeffrey K. Moore, Sarah Tan, Martin Poenie, James D. Lear, Tatyana Polenova, John A. Cooper, John C. Williams

**Affiliations:** 1 Department of Biochemistry and Molecular Biology, Thomas Jefferson University, Philadelphia, Pennsylvania, United States of America; 2 Department of Chemistry and Biochemistry, University of Delaware, Newark, Delaware, United States of America; 3 Department of Cell Biology & Physiology, Washington University in Saint Louis, Saint Louis, Missouri, United States of America; 4 Department of Cell and Molecular Biology, University of Texas, Austin, Texas, United States of America; 5 Department of Biochemistry and Biophysics, University of Pennsylvania, Philadelphia, Pennsylvania, United States of America; 6 Department of Molecular Medicine, Beckman Research Institute at City of Hope, Duarte, California, United States of America; Russian Academy of Sciences, Institute for Biological Instrumentation, Russian Federation

## Abstract

Cytoplasmic dynein and dynactin participate in retrograde transport of organelles, checkpoint signaling and cell division. The principal subunits that mediate this interaction are the dynein intermediate chain (IC) and the dynactin p150^Glued^; however, the interface and mechanism that regulates this interaction remains poorly defined. Herein, we use multiple methods to show the N-terminus of mammalian dynein IC, residues 10–44, is sufficient for binding p150^Glued^. Consistent with this mapping, monoclonal antibodies that antagonize the dynein-dynactin interaction also bind to this region of the IC. Furthermore, double and triple alanine point mutations spanning residues 6 to 19 in the yeast IC homolog, Pac11, produce significant defects in spindle positioning. Using the same methods we show residues 381 to 530 of p150^Glued^ form a minimal fragment that binds to the dynein IC. Sedimentation equilibrium experiments indicate that these individual fragments are predominantly monomeric, but admixtures of the IC and p150^Glued^ fragments produce a 2:2 complex. This tetrameric complex is sensitive to salt, temperature and pH, suggesting that the binding is dominated by electrostatic interactions. Finally, circular dichroism (CD) experiments indicate that the N-terminus of the IC is disordered and becomes ordered upon binding p150^Glued^. Taken together, the data indicate that the dynein-dynactin interaction proceeds through a disorder-to-order transition, leveraging its bivalent-bivalent character to form a high affinity, but readily reversible interaction.

## Introduction

Regulated, vectorial transport of signaling molecules, membranous organelles and cytoskeletal elements as well as the generation of forces to separate chromosomes and drive cellular motility are critical for cell function, viability and division [1]. Three classes of motor proteins, myosin, kinesin and cytoplasmic dynein, are responsible for force production and transport, and each produce force along cytoskeletal elements to coordinate these cellular processes. Myosins interact with actin filaments while kinesins and cytoplasmic dynein interact with microtubules [2]. Cytoplasmic dynein is unique in that as a family with a single member in mammals it is responsible for essentially all microtubule-based, retrograde transport [2], [3]. Cytoplasmic dynein, along with its regulatory partner dynactin, also participates in checkpoint signaling [4] and cell division [5] and requires both recognition and regulatory elements specific to each process. Point mutations and misfolding of dynein and/or its associated binding partners results in neurological diseases including spinal muscular atrophy (SMA), spinal and bulbar muscular atrophy (SBMA), amyotrophic lateral sclerosis (ALS), schizophrenia, and lissencephaly [6]–[8]. This further accentuates the critical role of cytoplasmic dynein, especially in highly polarized cells, such as motor neurons.

**Figure 1:**
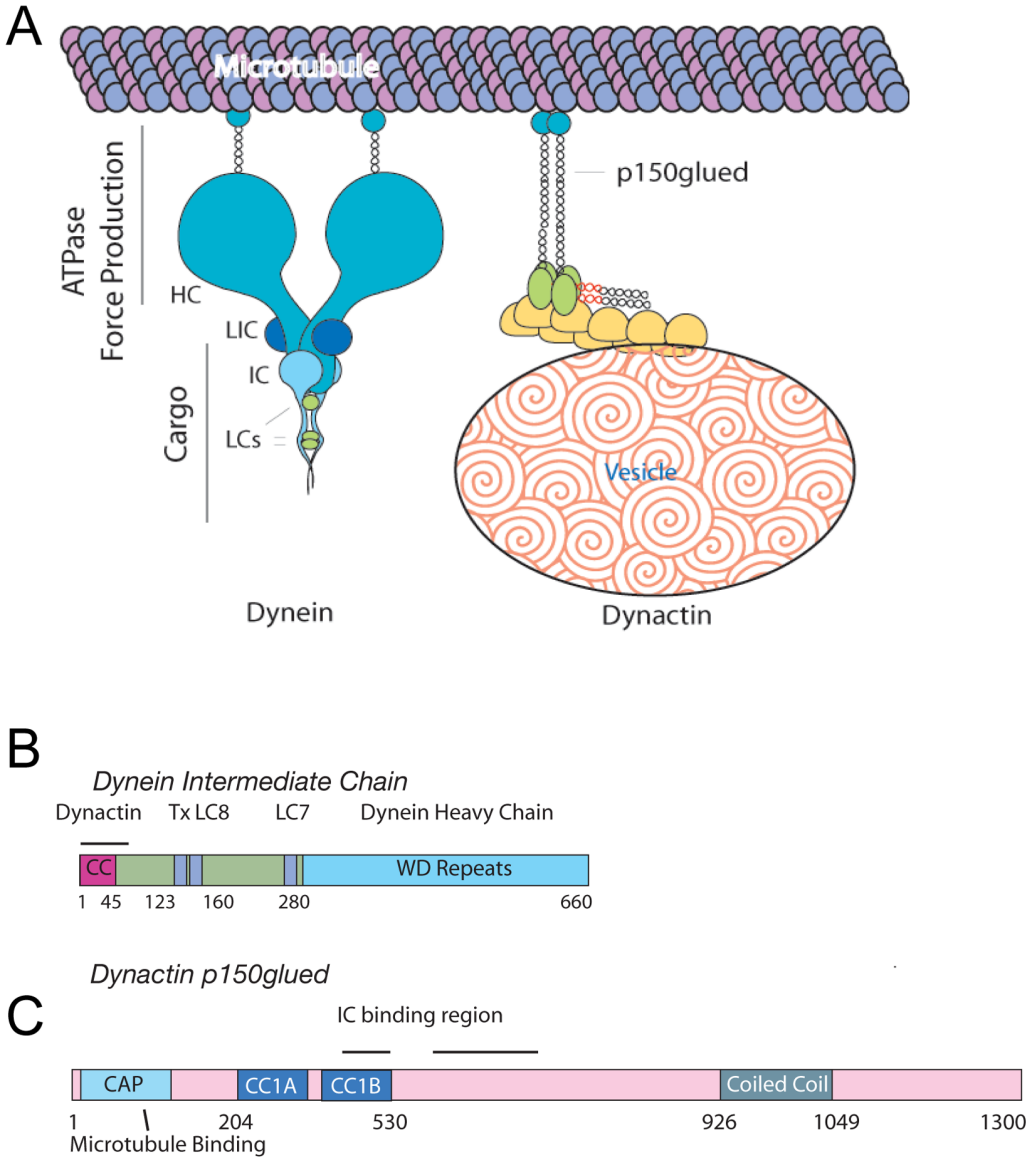
Dynein and dynactin schematic. *A*. Dynein and dynactin macromolecular complex organization. Both complexes are drawn approximately to scale from EM reconstruction images. *B*. Schematic of domain structure and binding sites of both the dynein intermediate chain isoform 2C and dynactin p150^Glued^. The dynactin binding domain, spanning residues 10–44 (indicated by a black line), is within the predicted N-terminal coiled-coil domain of the IC, residues 1–45. The intermediate chain binding interface has been mapped to fragments spanning residues 217–548, denoted CC1, or 600–811 (indicated by a black line).

Cytoplasmic dynein is a large, 1.2 MDa multisubunit complex that is composed of multiple homodimeric molecules (Fig. 1A). The heavy chain, 530 kDa, can roughly be divided into two functional regions. The C-terminus (residues ∼1140–4646, DYNC1H1) contains six AAA repeat domains and a coiled-coil stalk region responsible for microtubule binding. The AAA domains are responsible for force generation through ATP hydrolysis. The N-terminus of the HC (residues 1 to ∼1140) is dimeric and acts as a scaffold for both the dynein light intermediate chain (LIC) and the dynein intermediate chain (IC)(The residue numbering herein is based on the rat dynein and dynactin sequences, unless otherwise stated) [9], [10]. The dynein IC appears to contain two functional regions as well. The C-terminus contains a WD domain (residues 280–612, DYNC1I2 isoform C) that binds to the scaffolding region of the dynein HC. The N-terminal domain of the IC binds to the dimeric dynein light chains, TcTex1, LC8 and LC7 (residues 112–280). The extreme N-terminus of the IC (residues 1–106) binds to dynactin (Fig. 1B) [11]–[13]. Recently, Lis1 and NudE were also demonstrated to bind to the N-terminus of the IC as well [14], [15].

Dynactin was originally identified as a cytosolic activity that increases dynein's processivity along microtubules (Fig. 1A) [16]. Like dynein, dynactin is a large (1.2 MDa), multisubunit complex composed of eleven different oligomeric proteins [17]. A critical component of the dynactin complex is the homodimeric p150^Glued^. p150^Glued^ contains an N-terminal microtubule binding domain, the dynein IC binding site and a C-terminal scaffold for the vesicle binding components of the actin-like Arp-1 filament (Fig. 1B) [17].

Dynactin is required for reported dynein functions and disruption of this interaction affects cellular processes such as the mitotic checkpoint, Golgi positioning and retrograde transport [18]–[20]. While the mechanism that regulates the dynein-dynactin interaction is critical, little is known about it. In fact, the precise interface remains uncertain. Previous reports indicate the p150^Glued^ binding site on the dynein IC spans residues 1 to 106 of the IC (IC2C isoform) [11]–[13] and recent NMR results have further refined the dynactin interface to residues 1–87 [21]. This region contains a highly predicted coiled-coil, splice sites found in mammalian dynein [22], and a serine rich region. However, the IC binding site on p150^Glued^ is not clear. One report provides evidence that the dynein IC binds to a region spanning residues 217 to 548 of dynactin p150^Glued^ [13], [14], also strongly predicted to be a coiled-coil. In opposition with this, another report provides evidence that the IC binding site spans p150^Glued^ residues 600–811 [23], positioned within the predicted ARP1 rod binding site [17].

Beyond the conflicting results of the IC binding on p150^Glued^, there is also disagreement concerning how the interaction is regulated. Phosphorylation of dynein IC at Ser84 has been shown to block dynactin binding in blot overlay assays. However, expression of an N-terminal IC fragment containing the phosphomimic, Ser84Asp, did not affect dynein-mediated processes including endosome or Golgi dispersion. At the same time, the expression of the same fragment with a Ser84Ala mutation produced a range of dynein-mediated defects [24]. Even further confounding the issue, independent *in vitro* studies did not find any change in endomembrane localization upon expression of Ser84Asp, Ser84Ala or wildtype IC constructs [13].

To help resolve the discrepancies concerning both the interface and regulation of the dynein-dynactin interaction, and extend our recent studies suggesting that the dynein LCs regulate the interaction [25]–[27], we have generated a number of fragments of each component and used mutagenesis to refine the interface. In addition, we applied multiple methods to characterize the assembly of this critical interaction. We further confirm the importance of these interactions *in vitro* with cell-based assays. Collectively, these data suggest that residues 10–44 of the N-terminus of the dynein IC are sufficient to bind p150^Glued^ in a predominantly electrostatic interaction and that in doing so, they undergo a disordered-to-order transition. They also highlight the functional aspects of coupling disordered regions through a bivalent-bivalent interaction and provide additional support for a regulatory role of the LCs on dynein function [25]–[27].

## Materials and Methods

### Molecular Biology

*R. norvegicus* dynein intermediate chain isoform 2C (Accession number: AA8916; kindly provided by K. Vaughan, Notre Dame University) was used to amplify DNA for constructs spanning residues 1–124, 1–70, 1–44, 10–124, 20–124, 30–124 and 70–124. Primers for each are provided in the supplemental material. The IC DNA fragments were subcloned into the BamHI and XhoI sites of the pSMT3 vector (pET28 vector containing SUMO-1 homologprotein, kindly provided by C. Lima, Memorial Sloan Kettering) [28]. The vector produces a His6-SMT3-fusion protein (*S. cerevisiae* SUMO) resulting in increased expression of the fusion protein that is readily and specifically cleaved by ULP-1, a SMT3 specific protease [28]. Stop codons were engineered into SMT3-IC^1–44^ using standard site directed mutagenesis protocols to generate constructs spanning residues 1–18, 1–25, 1–32 and 1–39.

*R. norvegicus* p150^Glued^ (Accession number: NP_077044; also provided by K. Vaughan) was used to amplify DNA for constructs spanning residues 1–548, 204–530, 204–348, 381–530, 415–530 and 452–530. Primers for each are provided in the supplemental material. The p150^Glued^ DNA fragments were subcloned into the BamHI and HindIII or XhoI sites of the pSMT3 vector. A stop codon was engineered into p150^Glued^ 381–530 at residue 496 using standard site-directed mutagenesis. Stop codons were incorporated at residues 453, 476, 496 and 511 into the p150^Glued^ 415–530 construct following same protocol.

*S. cerevisiae* intermediate chain (*PAC11*) was amplified from yeast genomic DNA for a construct spanning residues 1–86. *PAC11* was subcloned into pGEX 6P-3 between the BamHI and XhoI restriction sites. Triple alanine mutations were introduced into *pac11* 1–86 at residues 4, 6, 9, 12, 14, and 19 and a double alanine mutant was introduced at residue 17 using standard site-directed mutagenesis protocols.

All constructs were verified by DNA sequencing.

### Protein Purification

All constructs were expressed in BL21 Star^TM^ (DE3) cells (Invitrogen) and induced at OD_600_  = 0.5–0.7 with 250 μM isopropyl β-D-1 thyogalactopyranoside for 3 hrs. The cells were pelleted, resuspended in 1× PBS (137 mM NaCl, 2.7 mM KCl, 4.3 mM Na_2_HPO_4_, 1.47 mM KH_2_PO_4_, pH 7.4) and stored at −20°C. The cells were lysed using a French press and clarified at 40,000 rpm at 4°C for 40 min.

SMT3-IC fusion proteins were isolated using Ni-NTA agarose (Qiagen) and subsequently incubated with Ulp1 enzyme [28] for 1–3 h at 4°C. Enzymatic activity was monitored by SDS PAGE (sodium dodecyl sulfate polyacrylamide gel electrophoresis). The buffer was exchanged to 50 mM Citrate pH 5.0, 1 mM dithiothreitol (DTT), 1 mM EDTA and protein was loaded onto a HiTrap™ SP XL column (GE Biosciences). The IC and SMT proteins were separated using a gradient from 100 mM to 800 mM sodium chloride over 100 ml. SDS PAGE was used to identify fractions containing the IC. Fractions were concentrated to 5 ml and loaded onto a HiLoad™ 26/60 Superdex 75 preparative column (GE Biosciences). The eluted protein was concentrated between 100 μM and 1 mM, aliquoted and stored at −80°C. For proteins containing an optical signal absorbance, 280 nm was used to determine concentration. For proteins without tryptophan, the final concentration was measured by total amino acid analysis. These results were subsequently correlated to the FluoroProfile® Protein Quantification Kit (Sigma).

SMT3-p150^Glued^ fusion proteins were isolated using Ni NTA agarose (Qiagen). The eluted protein was dialyzed against 50 mM Tris-HCl pH 8.0, 1 mM DTT, 1 mM EDTA and incubated with Ulp1 enzyme for 1–3 h at 4°C. Enzymatic activity was monitored by SDS PAGE. The protein was loaded onto a HiTrap™ Q XL column (GE Biosciences). The p150^Glued^ and SMT proteins were separated using a gradient from 100 mM to 800 mM sodium chloride. SDS PAGE was used to identify fractions containing p150^Glued^. p150^Glued^ was concentrated and loaded onto a HiLoad™ 26/60 Superdex 75 preparative column (GE Biosciences). The eluted protein was concentrated between 500 μM and 4 mM, flash frozen and stored at −80°C.

GST-Pac11 1–86 was isolated using Glutathione Sepharose™ 4B media (GE Biosciences). The eluted protein was incubated with precision protease (a gift from J. Pascal, Thomas Jefferson University) and dialyzed against 50 mM Tris-HCl pH 8.0 for two hours at room temperature. Cleavage of GST-Pac11 was confirmed by SDS-PAGE. GST was separated from Pac11 1–86 by running a second Glutathione Sepharose™ 4B column. The non-binding fraction was collected, concentrated to 5 ml and subsequently loaded onto a HiLoad™ 26/60 Superdex 75 preparative column (GE Biosciences). The eluted protein was concentrated between 200 μM and 1 mM, flash frozen and stored at −80°C.

### Analytical Size Exclusion Chromatography (SEC)

Equimolar amounts of the IC2B^1–158^-TcTex1-LC8 complex or p150^Glued^ (fragments CC1 – p150^Glued^ coiled-coil 1, CC1A – p150^Glued^ coiled-coil 1A and CC1B – p150^Glued^ coiled-coil 1B) were loaded on an analytical Superdex™ 200 10/300 GL column (GE Biosciences) alone or in combination. SDS-PAGE was used to verify protein content of eluted fractions.

Equimolar amounts of the Pac11-LC8 complex (either wild type or Pac11-R12A,Q13A,L14A) and CC1B were loaded onto an analytical Superdex™ 75 10/300 GL column (GE Biosciences) to assess binding. SDS-PAGE was used to verify protein content of eluted fractions.

### Sequence Comparison and Alignment

Intermediate chain sequences from yeast through human [Accession codes: AAP97254, AAA89165, NP_001069351, NM_068637, XM_685023, XM_855099, XP_001234410, XP_396853, AAL67574, NP_010776, BC086292.1, AF070697.1] and dynactin [Accession codes: NP_004073, NP_077044, XP_870398, XM_861321.1, NP_001080006, XP_700158.1, NP_001026538, NP_524061, XP_397370, NP_502033, AACD01000107.1, CAA51030] were aligned using ClustalW (see Fig. S1 A/B) [29]. Based on the alignment, sequences comprising the p150^Glued^ binding domain from both rat and yeast were analyzed with Align [30] to determine the sequence similarity of each region.

### Spindle position defects

Yeast manipulation, media and transformation were performed by standard methods [31]. Mutations in *pac11* were generated at the endogenous chromosomal locus, as previously described [31]. Mutations were confirmed by sequencing genomic loci. Expression of each *pac11* allele was confirmed by integrating a 13-myc epitope tag at the C-terminus, and assessing protein levels by anti-myc immunoblot. To assay for spindle positioning defects, cells expressing GFP-labeled microtubules (plasmid pSK1050, a gift from K. Lee at the National Institutes of Health) were grown to late log phase at 30°C, then diluted 1∶50 into fresh media and shifted to 12°C for 22 h. Defective spindle position was defined as cells containing either anaphase spindles entirely within the mother cell or supernumerary microtubule organizing centers.

### Circular Dichroism

CD spectra were collected with a J-810 spectropolarimeter (Jasco) using a 1nm bandwidth and 4 s response time. Molar ellipticity experiments were performed with 5 μM protein at 4°C using a 0.1 cm cuvette. Each experiment was performed in triplicate. Ellipticity is reported at mean residue ellipticity [θ] in deg cm^2^ dmol^−1^ using the equation: [θ]  = θ_obs_ x MRW/(10*l*c), where θ_obs_ is the observed ellipticity in degrees, MRW is the mean residue weight (molecular mass/number of residues), *l* is the path-length in cm, and c is the concentration in mg/ml. The mean residue ellipticity for a 100% helical protein was calculated from the equation [θ]_222_  = 40×10^3^×(1–4.6/n) where n equals the number of residues in the protein [32]. The helical percentage of each protein was determined from the ratio of [θ]_222_ experimental divided by the [θ]_222_ theoretical value.

Temperature scans were performed with 5 μM for individual protein scans or equimolar amounts of 3.5 μM of each protein in a 1 cm cuvette with constant stirring from 0–80°C. The wavelength was recorded at 222 nm with a data pitch of 1°C, a 45 s delay, and a temperature slope of 40°C/h. The melting point (T_M_) of each protein was calculated from the first derivative of the data. Each experiment was performed in triplicate.

### Native PAGE

Native PAGE analysis was performed using both Mini 12% cross-linked gels with a stacking gel at pH 7.4 or 8–25% gradient gels. Purified proteins were incubated at 25°C for five minutes in equimolar or two fold molar excess to assess binding characteristics.

### Western Blotting

2 ml cultures of each indicated SMT3-IC fusion construct were expressed at 37°C for 3 h. Cells were harvested and the resulting pellet was resuspended in 100 μl SDS loading dye. Each (20 μl) overexpression was loaded onto two 15% SDS PAGE gels. Overexpression was confirmed by Coomassie staining. Proteins were transferred to a nitrocellulose membrane using the submersed method. The membrane was blocked using 5% (w/v) dry milk in PBS-T (1× PBS.05% Tween 20). IC constructs were detected using either mouse monoclonal anti-IC 70.1 or mouse monoclonal anti-IC 74.1 (a gift from K. Pfister, University of Virginia). For visualization, blot membranes were incubated with goat anti-mouse secondary conjugated to horseradish peroxidase (Sigma) and developed using Pierce ECL western blotting substrate.

### Sedimentation Equilibrium Analysis

Temperature dependence of binding was determined using sedimentation equilibrium performed at either 4 or 20°C using the Beckman-Coulter XLI Analytical Ultracentrifuge. Samples of individual proteins or protein complexes were dialyzed against 1× PBS and 1 mM TCEP. Protein concentrations were analyzed between 0.1–0.6 OD_280/230nm_ using three or more speeds. Equilibrium was assessed by radial scans at both 10 and 12 h during each speed. Data were analyzed using conventional equilibrium equations incorporated into IgorPro (Wavemetrics) [33]. The partial specific volume for each construct was calculated using SEDNTERP [34]. Values are as follows: CC1  = 0.721, CC1A  = 0.727, CC1B  = 0.726, IC^1–44^  = 0.736 and IC^1–124^ = 0.731. Extinction coefficient for 230 nm was calculated at 900 absorbance units per residue. For molecular weight determinations, v_BAR_, ρ (density), and ε (extinction coefficient) were kept constant, the protein concentration was allowed to float, while the molecular weight was calculated. It is important to note the reported errors for molecular weight are from the Fast Fitter. Instrumental error is ±5%. For association constants, v_BAR_, ρ, ε, n (number of associating units), molecular weight and concentration were kept constant, while the association constant was fit.

Dimerization and temperature dependence assays were performed using p150^Glued^ (CC1 or CC1B) and IC^1–124^ individually and as admixtures. A single speed of 25000 rpm was analyzed at 5, 10, 15, 20 and 25°C. Each speed was fit to a single molecular weight to assess; 1) oligomeric state of IC or p150^Glued^ and 2) complex formation between IC and p150^Glued^.

pH and salt dependence assays were performed on both IC^1–124^ and p150^Glued^ CC1 individually and in complex at 4°C. Four speeds were analyzed for each component over a range of pH 6.0–9.0 and sodium chloride concentrations from 0–1 M.

### Cross-linking Experiments

3,5 dimaleimidobenzoic acid (3,5 DMBA) was synthesized as previously described (assisted by Prof. David Horne and Yuelong Ma, City of Hope) [35]. A linker, CysGlyGlySer, was engineered at the N-terminus of p150^Glued^ CC1 using standard subcloning protocols. Cys-CC1 was expressed and purified as described above. Cys-CC1 was exchanged under argon into 20 mM phosphate buffer pH 7.4 1 mM TCEP. 50 μM Cys-CC1 was incubated with 50 μM 3,5 DMBA for one hour. Samples were run on an 8% SDS PAGE gel under non-reducing or reducing conditions to assess cross-linking.

## Results

### p150^Glued^ binding site on the IC

**Figure 2:**
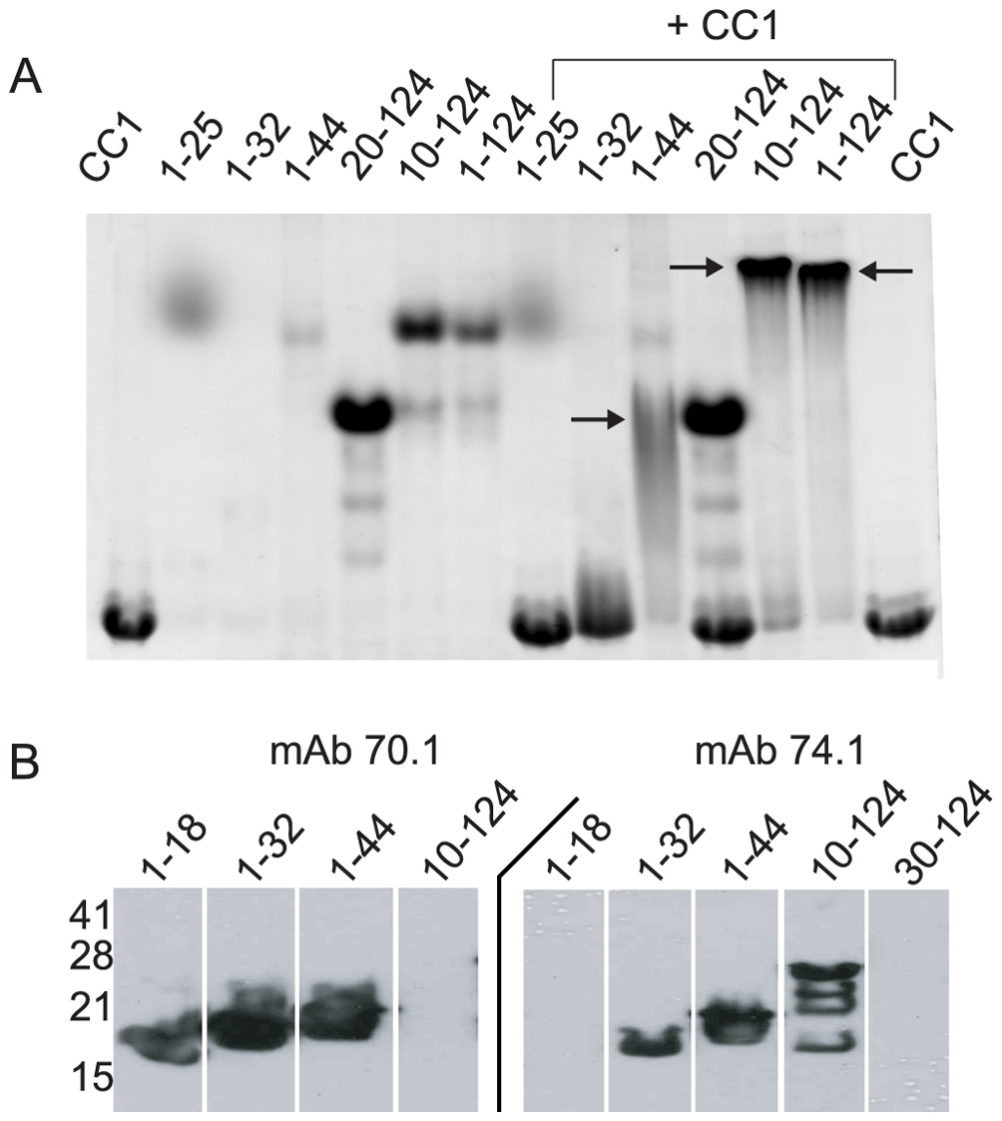
Dynein intermediate chain minimal binding domain. *A*. Native PAGE indicates that residues 10–44 are sufficient for binding to p150^Glued^ CC1. A gel shift indicates IC2C fragments spanning residues 1–124, 10–124 and 1–44 are capable of binding CC1 (indicated by arrows). However, fragments spanning 20–124 and 1–32 are not able to bind to CC1. It is important to note that due to the large negative charge of the IC some constructs to not enter the gel. *B*. Epitope mapping of IC antibodies: The epitopes of α-IC mAb 70.1 and 74.1 are located within the p150^Glued^ binding domain. Specifically, α-IC 70.1 recognizes the region between residues 1–18 and α-IC 74.1 recognizes 10–30.

The IC and p150^Glued^ fragments contain predicted coiled-coil regions, suggesting that this interaction may form a hetero-tetrameric coiled-coil (Fig. 1B). While previous studies report that the minimal region of the IC capable of binding p150^Glued^ spans residues 1 to 106, only the first 44 residues of the N-terminus of the IC in mammalian sequences are strongly predicted to form a coiled-coil (Fig. S2A) and are highly conserved across multicellular organisms. The region between the predicted coiled-coil and residue 106, on the other hand, is highly variable and not conserved. To discriminate whether the coiled-coil region is sufficient to bind to p150^Glued^ or residues to the C-terminus are required, we generated a series of constructs truncated at either the N-terminus or C-terminus of the IC. Native PAGE, a non-denaturing gel electrophoresis method that separates protein based on charge and hydrodynamic size, was used to determine the ability of IC fragments to bind to p150^Glued^. We note that the IC fragments and p150^Glued^ fragments are highly, but oppositely charged. Thus, several of the IC constructs migrate to the opposite pole and are not observed in the gel, but if there is an interaction, the overall charge is reduced and the complex will migrate into the gel. We observe IC constructs encoding residues 1–44 and 10–124 bind to p150^Glued^ (see below), but 1–25 and 20–124 do not (Fig. 2A). The construct, 1–32, produces a smear, suggesting weak affinity. Collectively, these data show residues 10–44 of the IC are sufficient to bind to p150^Glued^. This mapping is consistent with recent NMR studies using the *Drosophila* sequence [21].

Based on this mapping, we tested if IC antibodies, 70.1 and 74.1, used to disrupt the dynein-dynactin interaction in microinjection experiments, map to the same region of the IC (Fig. 2B) [36].- Using the truncated constructs, we observe that mAb 70.1 binds to residues 1 to 18, but not residues 10–124, of the IC, indicating that the epitope of mAb 70.1 lies between residues 1–18. mAb 74.1 binds to the IC construct spanning residues 10–124, but not to constructs spanning residues 1–18 or 30–124, indicating that the epitope for mAb 74.1 lies between 10 and 30. These results indicate that both antibody epitopes overlap with the p150^Glued^ binding site on the IC, consistent with their role in disrupting the dynein-dynactin interaction through competition. It should be noted that using either antibody to immunoprecipitate dynein invariably fails to pull down dynactin components [37], [38].

### Alanine Scanning Mutagenesis of Dynein IC

**Figure 3:**
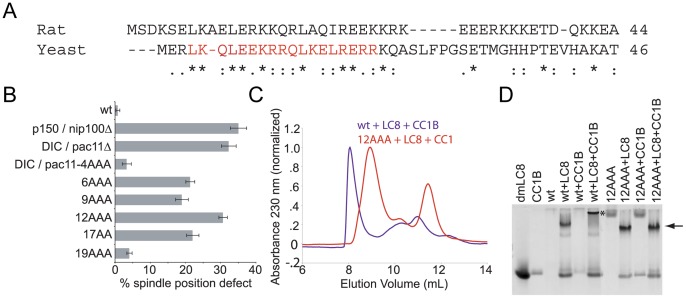
Alanine scanning mutagenesis of Pac11. *A*. Sequence alignment of R. norvegicus IC and S. cerevisiae Pac11 (IC). Alanine point mutations were introduced into Pac11 (indicated in red). A (*) indicates identical amino acids, (∶) indicates highly conserved, similar amino acids, (.) indicates amino acids that are somewhat similar and blank indicates dissimilar amino acids. *B*. Native PAGE indicates that *S. cerevisiae* wt-Pac11^1–86^ is capable of binding p150^Glued^ CC1B alone (indicated by the loss of CC1B) and in the presence of dynein light chain 8 (LC8) (indicated by the gel shift of LC8). Due to the charge and hydrodynamic properties of wt-Pac11^1–86^, it does not enter the native PAGE. An asterisk indicates gel shift upon wt-Pac11^1–86^-LC8-CC1B binding. Pac11^1–86^ triple point mutant Pac11-R12A,Q13A,L14A (12AAA) is unable to bind p150^Glued^ CC1B alone or in the presence of LC8. Arrow indicates formation of Pac11-R12A,Q13A,L14A-LC8 (12AAA) binding. No gel shift occurs upon addition of CC1B. *C*. Size exclusion chromatography indicates wt-Pac11^1–86^ forms a complex with LC8 and CC1B (purple), with an elution volume of 8.07 mL, while Pac11-R12A,Q13A,L14A (12AAA) is unable to bind to CC1B (red). The Pac11-R12A,Q13A,L14A(12AAA)-LC8 complex elutes at 8.97 mL and CC1B elutes at 11.45 mL. *D*. Spindle positioning in wild-type and mutant cells expressing GFP-labeled microtubules. The percentage of cells exhibiting spindle position defects (see Materials and Methods) was determined for wild-type (yJC5919), p150^ Glued^/*nip100*Δ (yJC6047), DIC/*pac11*Δ (yJC6354), DIC/*pac11-*4A (L4A,K5A,Q6A, yJC6916), *pac11-*6A (Q6A,L7A,E8A, yJC6917), *pac11-*9A (E9A,K10A,R11A, yJC6918), *pac11-*12A (R12A,Q13A,L14A, yJC6846), *pac11-*17A (L17A,R18A, yJC6847), and *pac11-*19A (E19A,R20A,R21A, yJC6919) strains. Error bars denote SEM. P-values are shown in Fig. S3B.

As a complementary and biologically relevant analysis, we characterized the ability of the *S. cerevisiae* dynein intermediate chain (Pac11) to bind to p150^Glued^ *in vitro* and *in vivo* based on the mapping studies above. The N-terminal predicted coiled-coil of mammalian and yeast IC are 22.4% identical and 42.9% similar (overall, IC and Pac11 are 17.6% identical and 30.2% similar) and have a high proportion of negatively charged amino acids (Fig. 3A). First, we established that the wild type Pac11 N-terminal fragment, residues 1 to 86, which contains two dynein LC8 binding sites [27], binds to mammalian p150^Glued^ by sedimentation equilibrium measurements of a complex of p150^Glued^, Pac11 and LC8. This fits to a molecular weight of 102,300 ±300 Da (data not shown), consistent with the theoretical mass of the complex being 97,108 Da. Next, mitotic spindle positioning assays in *S. cerevisiae* were used to assess dynein function. In these studies sequential double and triple alanine mutations were generated in *PAC11* and expressed using the *PAC11* endogenous promoter. Shown in Fig. 3B and S3A, the percentage of cells with either long anaphase spindles or multiple microtubule organizing centers (MTOCs) located within the mother cell was determined for wild-type, *nip100*Δ, *pac11*Δ, *pac11*-L4A,K5A,Q6A (4AAA), *pac11*-Q6A,L7A,E8A (6AAA), *pac11*-E9A,K10A,R11A (9AAA), *pac11*-R12A,Q13A,L14A (12AAA), *pac11*-L17A,R18A (17AA), and *pac11*-E19A,R20A,R21A (19AAA) strains. Defects are observed for point mutations extending from 6 to 18, with the triple point mutant, *pac11*- R12A,Q13A,L14A showing nearly the same phenotype as completely removing either Pac11 or Nip100, the yeast p150^Glued^ homolog. The complete loss of these genes produces the strongest phenotype.

Subsequently, biochemical analysis was used to confirm that spindle positioning defects were a direct result of abrogation of the Pac11-p150^Glued^ interaction. Triple and double alanine Pac11 point mutants were generated, purified to homogeneity, and their affinity for p150^Glued^ was tested by native PAGE and size exclusion chromatography. The N-terminal fragment of p150^Glued^ CC1, denoted CC1B, was used for this analysis (see explanation in subsequent section). Consistent with *in vivo* results, the triple point mutant Pac11-R12A,Q13A,L14A (12AAA) has the largest effect on p150^Glued^ binding. Both native PAGE and analytical gel filtration indicate that the triple point mutation Pac11-R12A,Q13A,L14A (12AAA) abrogates the Pac11-p150^Glued^ interaction in the presence or absence of LC8 (Fig 3C/D). On the other hand, mutants Pac11-L4A,K5A,Q6A (4AAA), Pac11-Q6A,L7A,E8A (6AAA), Pac11-E9A,K10A,R11A (9AAA), Pac11-L17A,R18A (17AA), and Pac11-E19A,R20A,R21A (19AAA) fail to bind p150^Glued^ in the absence of LC8. However, in the presence of LC8, the latter point mutants also bind p150^Glued^ (Fig. S3B). Collectively, these results further confirm that the extreme N-terminus of the IC is necessary for the dynein-dynactin interaction. Moreover, they also indicate that critical elements of the IC-p150^Glued^ interaction are conserved across species.

### IC binding site on p150^Glued^

The IC binding site on p150^Glued^ remains unclear. Initial studies show that a p150^Glued^ fragment spanning residues 1 to 811 is sufficient to bind the IC [12], but subsequent refinement indicated that the IC binds to p150^Glued^ fragments either spanning residues 217 to 548 [13] or 600 to 811 [23]. The difference in the mapping produced in these studies could be due to different techniques used to detect binding (e.g., blot overlay versus solution-based methods) or differences in experimental conditions (e.g., buffers, temperature, pH, salts). In addition, the purification tags used in these studies differed both in composition and location. In order to move forward in characterizing the dynein-dynactin interface, we first attempted to resolve this difference by producing multiple constructs of mammalian p150^Glued^, removing the purification tag and using multiple methods to characterize the binding.

First, we generated the same fragments previously published and used the histag-SMT3 fusion to produce and purify each fragment. Using a recombinant histag-Ulp1 protease, the histag-SMT3 fusion was readily and specifically cleaved from the p150^Glued^ fragments. It is important to point out that the cleavage leaves a single residue, serine, at the N-terminus of the p150^Glued^ fragment. We note that the N-terminal histag-SMT3 fusion dramatically increases the expression of different constructs compared to a N-terminal histag only. We observe that the p150^Glued^ fragment, residues 1–548, was readily soluble whereas p150^Glued^ fragments encoding residues 1–811 and 600–811 were insoluble (data not shown). A number of attempts were made to solubilize the second site including altering expression temperature, inducing chaperones, using different cell lines, and refolding the protein from inclusion bodies, but well-behaved (soluble) protein was undetected.

**Figure 4:**
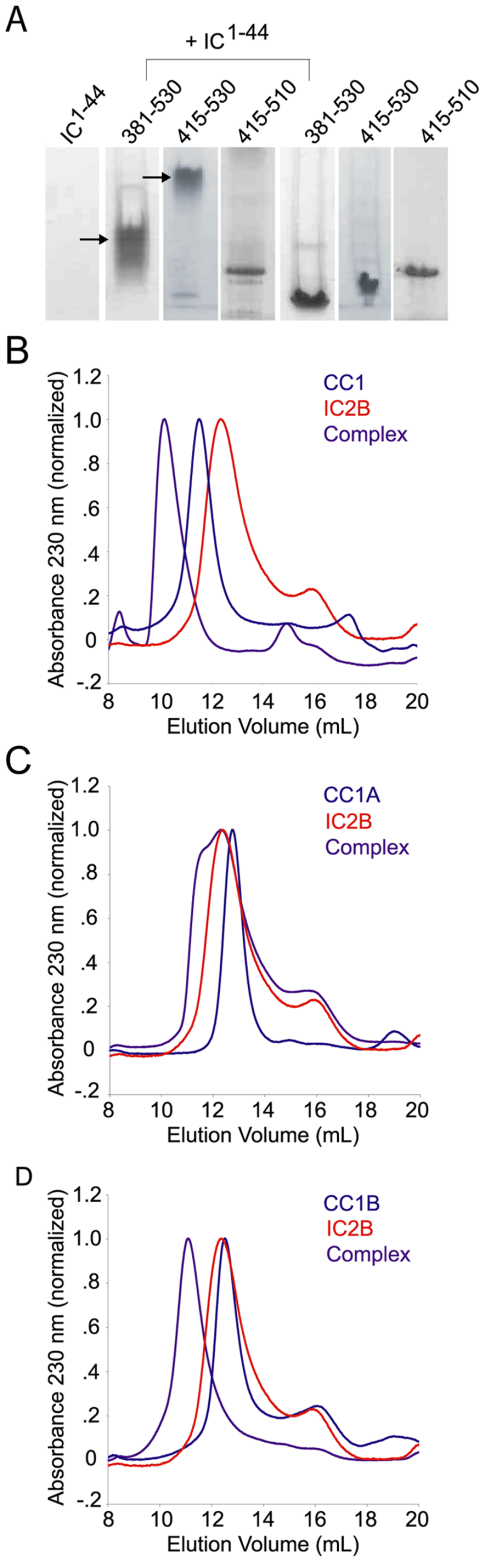
p150^Glued^ minimal binding domain. *A*. Native PAGE indicates that residues 381–530 and 415–530 of p150^Glued^ are sufficient to bind to the intermediate chain as shown by the gel shift (indicated by arrows) upon incubation of p150^Glued^ with IC^1–44^. Each binding experiment, p150^Glued^ fragment + IC, was performed on an individual gel. Lanes are representative of individual binding experiments. *(B–D)* Size exclusion chromatography of p150^Glued^ fragments in complex with IC2B^1–158^-TcTex1-LC8. *B*. CC1, IC2B^1–158^-TcTex1-LC8 alone or in complex. *C*. CC1A, IC2B^1–158^-TcTex1-LC8 alone or in complex. *D*. CC1B, IC2B^1–158^-TcTex1-LC8 alone or in complex. The change in elution volume of the IC complex is 2.22, 0.08 and 1.26 ml in the presence of CC1, CC1A and CC1B, respectively.

Consequently, we focused our efforts on the N-terminal region, residues 1 to 548. Based on coiled-coil predictions, previous studies have generated a construct that spans 217 to 548 and denoted CC1 [13]. Moreover, the N-terminal region of the IC has been strongly predicted to contain a coiled-coil region in the first 44 residues (see previous section). We reasoned that if the interaction proceeds through a coiled-coil, CC1 should contain a subdomain that binds to the predicted 6 heptad repeats of the IC. First, we used limited proteolysis to identify stable boundaries within the p150^Glued^ fragment spanning 1 to 548. We observed a stable fragment of approximately 32 kDa and used N-terminal sequencing to show that this fragment starts at residue 204 (data not shown). We observed a slight degradation at the C-terminus corresponding to approximately a 2 kDa difference. These results indicate that residues 204 to 530 are stable to limited proteolysis. Using sequence alignment and coiled-coil prediction algorithms [29], [39], we observe a conserved break spanning residues 349 through 380 in the coiled-coil fragment, across multiple species (yeast to human; Fig. S2B). To test which fragment encompasses the IC interface, we generated two new fragments of CC1, denoted CC1A (residues 204–348) and CC1B (residues 381–530). Surprisingly, gel filtration experiments indicate that both CC1A and CC1B interact with dynein IC2B/C pre-saturated with the dynein light chains, LC8 and TcTex-1 (residues 1 to 158 of 2B and 1 to 124 of 2C) (Fig. 4). On closer analysis, the size exclusion data show a significant shift in the elution volume of an admixture of CC1B and IC fragment bound to the dynein light chains TcTex1 and LC8 compared to their individual elution volumes. Little or no change in elution volume is observed in the admixture of CC1A and the IC complex; however, SDS-PAGE of the fractions corresponding to the CC1A elution volume shows the presence of the IC. We note that the IC and CC1 are highly, but oppositely charged (Fig. S2 C/D). Thus, we suspect that the interaction between the dynein IC and CC1A or CC1B using these methods is partially due to electrostatic effects (see below).

Based on SEC results and results presented below, we further truncated the CC1B fragment. Expecting that the IC-p150^Glued^ interaction forms a hetrotetrameric coiled-coil, new p150^Glued^ fragments were made by systematically deleting predicted heptads from the N- and C-terminus of CC1B. Native PAGE experiments indicate residues 415–530 of p150^Glued^ are sufficient to bind to IC^1–44^ (Fig. 4A). However, the interaction is lost upon further truncation of either ten or three heptad repeats at the N- and C-terminus of CC1, respectively. Specifically, constructs spanning residues, 381–495, 415–510 and 489–530 did not produce a shift (Fig. 4A and data not shown). Residues 415–530 contain 16 potential heptad repeats.

### Biophysical Characteristics of the Individual Binding Domains

**Figure 5:**
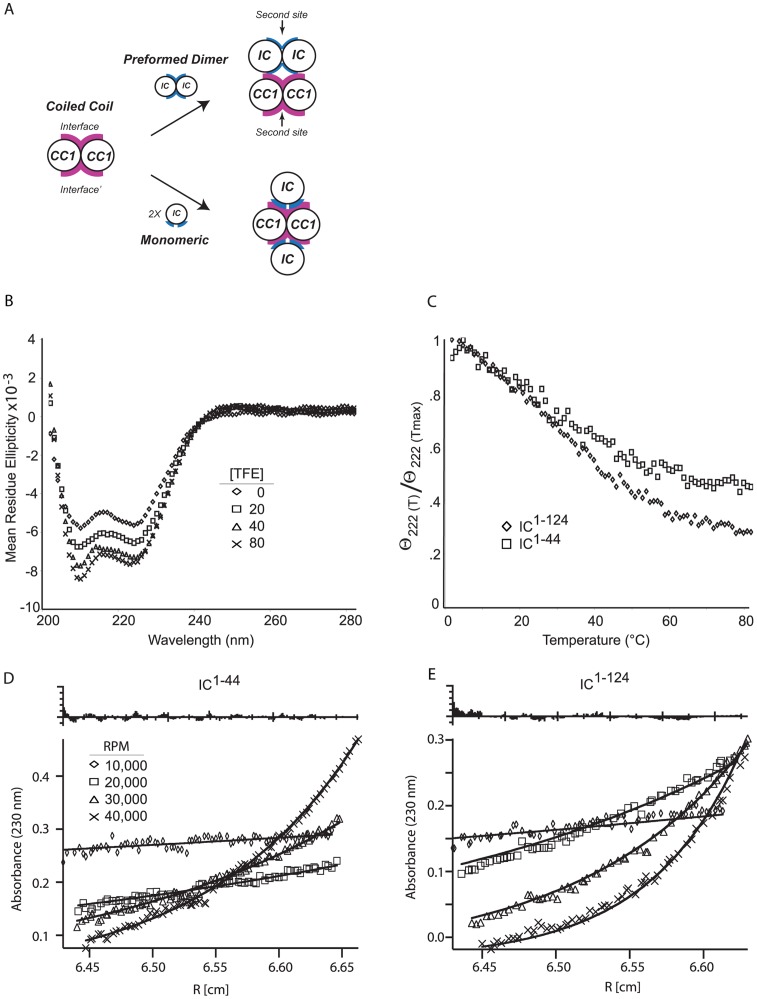
Biophysical characteristics of intermediate chain fragments. *A.* Cartoon of two potential mechanisms to create a dimer-of-dimers through a coiled coil. Magenta highlights the IC binding interface on CC1. Blue indicates the CC1 binding site on the IC. The top route assumes that the IC is homodimer before binding to CC1. The bottom route assumes that the IC is monomeric before binding the CC1. *B*. Circular dichroism spectra of 20 μM IC^1–44^ in 1− PBS 1 mM TCEP at 4°C with increasing concentrations of trifluoroethanol results in a minimal increase of α–helical characteristics. *C*. Thermal denaturation of 20 μM IC^1–44^ and IC^1–124^. Thermal denaturation was monitored at 222 nm. *D/E*. Sedimentation equilibrium: sedimentation equilibrium analytical ultracentrifugation (SE-AUC) at 10000, 20000, 30000, 40000 rpm and 20°C show that both IC^1–44^ (*C*) and IC^1–124^ (*D*) are monomeric.

Based on the observation that all dynein subunits and the dynactin p150^Glued^ subunit are predicted to be homodimeric [3], we hypothesized that the IC and p150^Glued^ fragments interact through a local symmetry axis, forming a hetero-tetrameric complex, or a ‘dimer of dimers’, which implies that the regions comprising the interface of one or both components must be monomeric in the absence of the other preceding the interaction. This hypothesis stems from ‘symmetry’ considerations and is supported by multiple crystal structures of ‘dimer of dimers’ interactions (e.g., transcription factors bound to palindromic DNA [40], the IC peptide binding to LC8 [41], EB1 binding to the CAP-gly domain of p150^Glued^ [42], etc.). Specifically, in forming a homodimeric coiled coil, two binding surfaces are created (see Fig. 5A). To form a 2∶2 complex, the interacting partner would have to bind to both surfaces, otherwise an oligomer would form. To do this, either p150^Glued^ or the IC would have to be monomeric. Otherwise, it is possible to create an oligomeric species (see Fig. 5A).

To test this, we used sedimentation equilibrium analytical ultracentrifugation (AUC), and circular dichroism (CD) experiments to quantify the hydrodynamics and secondary structure properties of each individual component. Molar ellipticity curves (Fig. 5B) of this fragment indicate a minimal amount of α-helical structure, as illustrated by the slight minima seen at both 222 and 208 nm. The θ_222 nm_ of −5.7 deg cm^2^ dmol^−1^ corresponds to approximately 14% helicity or 6 residues (a θ_222 nm_ value of −35.8 deg cm^2^ dmol^−1^ would correspond to a 100% helical peptide of 45 residues). To test the propensity of residues 1–44 to exist in an α-helix we incubated IC^1–44^ with increasing concentrations of trifluoroethanol (TFE), previously shown to induce helical structure in peptides with the propensity to form a helix. Upon addition of up to 80% TFE we see little change in the α-helical content of the IC^1–44^, an increase from 14% to 21% or 9 residues total. This suggests that the IC fragment is not intrinsically helical. In addition, thermal denaturation of both IC^1–44^ and IC^1–124^ show no transition, further confirming these fragments are not folded (Fig. 5C). Finally, there is no concentration dependence seen in the CD spectra of the IC (data not shown).

Next, we used sedimentation equilibrium to determine the oligomeric state of both IC^1–44^ and IC^1–124^ (Fig. 5 D/E). Sedimentation equilibrium experiments were performed at 10000, 20000, 30000, and 40000 rpm at 20°C. Using FastFitter, the calculated molecular mass of IC^1–44^ is 6100±200 Da (Red. χ^2^ = 2.9) [33]. The theoretical weight is 5470 Da. Similar measurements were made for the IC^1–124^ fragment to rule out potential dimerization values from the residues between the predicted coiled-coil region and the TcTex1 binding site. The calculated molecular weight of this construct, IC^1–124^, is 13,200±10 Da, which is consistent with the theoretical monomeric weight of 13,880 Da. Taken together, these data indicate that the N-terminal region of the IC is monomeric and disordered.

**Figure 6:**
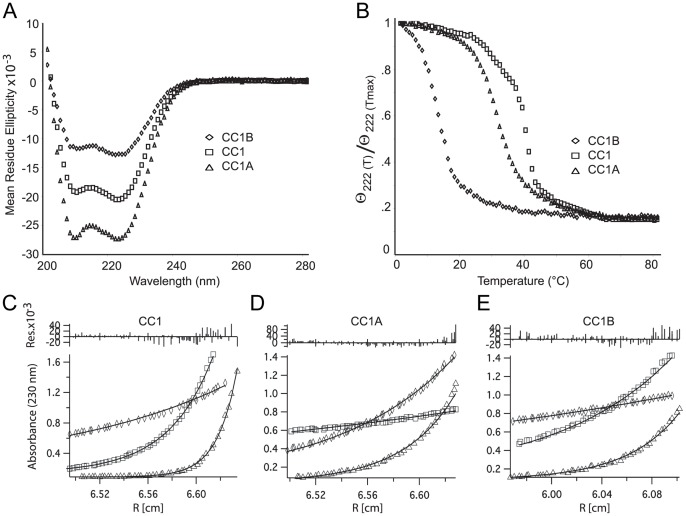
Biophysical characteristics of p150^Glued^ fragments. *A*. Circular dichroism spectra of 5 μM p150^Glued^ CC1, CC1A and CC1B fragments in 1 x PBS 1 mM TCEP indicate they are predominantly helical at 4°C. *B*. Thermal denaturation of 10 μM p150^Glued^ CC1, CC1A and CC1B. Thermal denaturation was monitored at 222 nm. *C–E*. Sedimentation equilibrium: SE-AUC at 20000, 25000, 30000, 35000 rpm and 4°C show that all three fragments, CC1 (25000, 30000 and 35000 rpm only) (*C*), CC1A (*D*) and CC1B (*E*) are dimeric.

As with the IC fragments, CD and sedimentation equilibrium measurements were used to quantify the secondary structure content and oligomeric state of the p150^Glued^ fragments. CD spectra of the p150^Glued^ fragments indicate that each construct is predominantly α-helical at 5 μM (Fig. 6A), with molar ellipticity values of −20.5, −27.5 and −12.7 deg cm^2^ dmol^−1^, for CC1, CC1A and CC1B, respectively. The CC1A fragment has a high percent helicity, 73%, whereas the CC1B fragment is lower, 31%. The percent helicity of the full-length CC1 fragment is 52%. This value is the geometric mean of CC1A and CC1B, indicating that the presence of the CC1A helix does not increase the propensity for CC1B for forming a helix (100% α-helical character of CC1, CC1A and CC1B would produce a signal of −39.4, −38.7 and −38.7 deg cm^2^ dmol^−1^, respectively). Thermal denaturation experiments, performed by monitoring the CD signal at θ_222 nm_ while increasing the temperature, show that CC1, CC1A and CC1B denature at 38°C, 27°C and 13°C, respectively (Fig. 6B). Each experiment was done in triplicate and the error for each is ±1°C. In these experiments, we observe that CC1A melting is reversible, but not CC1 or CC1B (data not shown).

Next, we measured the hydrodynamic properties of CC1, CC1A and CC1B using sedimentation equilibrium experiments. Since CC1B has a melting point of 13°C, we collected sedimentation equilibrium data at 4°C and observed an increase in the mass and poor fit assuming a single species. Thus, we fit the data to a monomer-dimer model and observed a dimer dissociation constant (K_d_) <60 nM (Fig. 6E). Sedimentation equilibrium studies were also collected at 4°C for CC1 and CC1A (Fig. 6C/D). Both proteins also form dimers at this temperature with estimated K_D_'s <1 and <40 nM, respectively (fitting analysis in Fig. S4A).

When comparing our CD and AUC data to recent electron microscopy images (See Fig. 1C in Imai [43]), we propose that CC1A constitutes the “arm” of the dynactin complex. Specifically, the CD spectrum of the p150^Glued^ fragments, CC1A and CC1B, indicates the percent helicity of these constructs are 73% and 31%, respectfully. Given that α-helices rise 1.5 Å/residue and CC1A is 145 residues, we calculate that the end-to-end length of CC1A is 154 Å. On the other hand, CC1B is 149 residues with 30% helical content suggesting an end-to-end distance of 67 Å. Based on EM projections in Imai (2006), we calculate the average length of the dynactin arm to be 148±22 Å (5 images) [43]. This distance is comparable with an extended coiled-coil structure of CC1A. In addition, we engineered cysteine residues at the N-termini of CC1 and show that it is rapidly crosslinked by 3,5 DMBA (Fig. S5). Also, using a split fluorescein arsenical hairpin (e.g., a FLaSH tag with two cysteines at the N-termini of CC1), we observe a fluorescent signal upon the addition of the FLaSH reagent but not for the non-modified CC1 (data not shown) [44]. While not unexpected, these data provide experimental evidence that CC1A forms a parallel coiled-coil.

### Stoichiometry and Energetics of the Dynein-Dynactin Interaction

**Figure 7:**
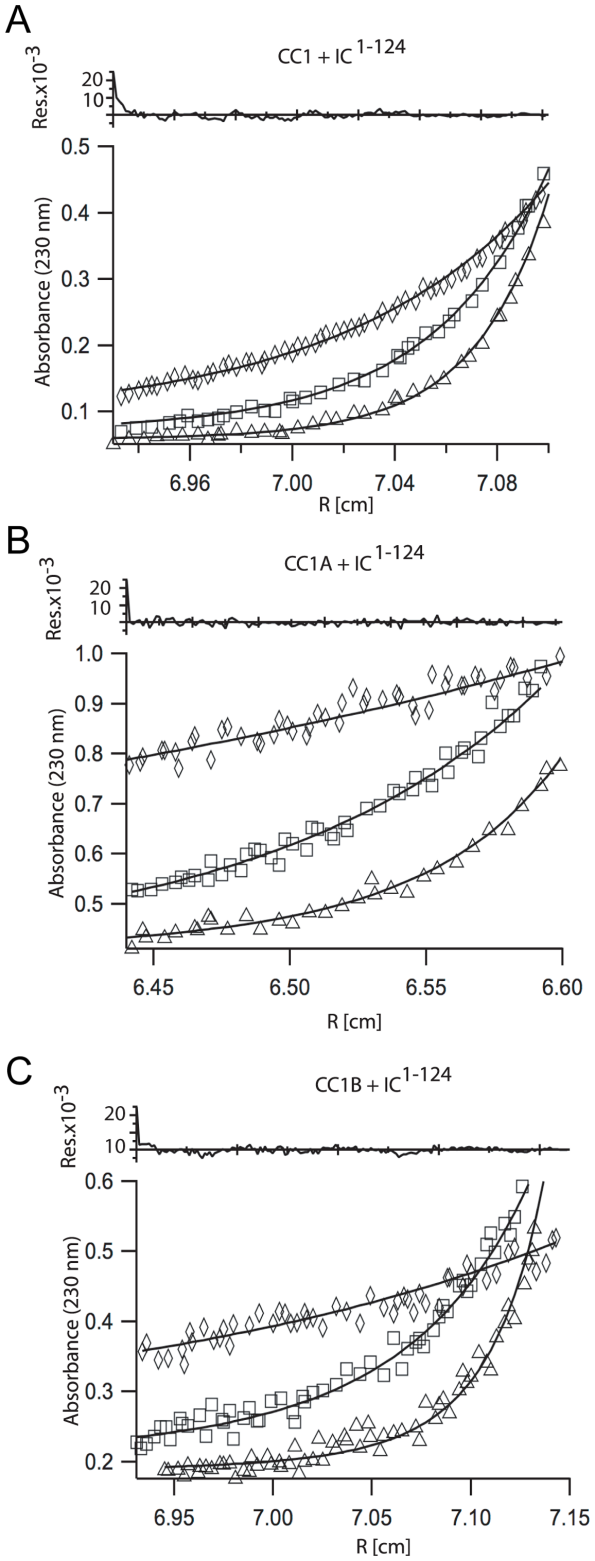
Stoichiometry of the IC-p150^Glued^ interaction. *A–C*. Sedimentation equilibrium: *A.* SE-AUC at 12000, 16000 and 20000 and 4°C of CC1 and IC^1–124^. *B–C.* SE-AUC at 10000, 20000, and 30000 rpm and 4°C of CC1A (B) and CC1B (C) with IC^1–124^. Both CC1 and CC1B associate much more strongly with IC^1–124^ than CC1A.

All components of dynein and the p150^Glued^ subunit of dynactin are dimeric within the assembled complexes. Thus, we and others propose that the IC-p150^Glued^ interaction proceeds through a heterotetrameric complex. Sedimentation equilibrium experiments at 4°C indicate both CC1 and CC1B p150^Glued^ fragments associate with IC^1–124^ (Fig. 7A/C). Sedimentation equilibrium experiments at 4°C of CC1 and CC1B indicate they are dimeric based on the calculated dissociation constants, therefore the data were fit to a model of CC1_DIMER_ +2 IC fragments → CC1_DIMER_-IC_2_ with a dissociation constant <10^−15^ M^2^ (N.B., the units are molar squared since we treated the IC as individual fragments). CC1B was fit to a model of CC1B_DIMER_ +2 IC → CC1B_DIMER_–IC2 with a dissociation constant between 10^−11^ and 10^−13^ M^2^. The same analysis applied to AUC measurements on a stoichiometric admixture of 10 μM CC1A and 10 μM IC1−124 (Fig. 7B) showed little or no heteromeric association (dissociation constant >10^−10^ M^2^). See fitting analysis in figure S4B.

Complementary binding analysis was performed using thermal denaturation experiments with equimolar (3.5 μM) amounts of p150^Glued^ fragments, CC1A or CC1, and IC^1–44^. No increase in melting temperature occurs upon incubation of CC1A and IC^1–44^, compared to CC1A alone (Fig. S6A). In contrast, thermal denaturation of CC1B and IC^1–44^ indicates a melting temperature of 16±1°C, a three degree increase from CC1B alone (Fig. S6B). Both sedimentation equilibrium and thermal denaturation data indicate that at concentrations below 10 μM CC1A does not associate with the IC.

Taken together, sedimentation equilibrium and thermal denaturation data of CC1B indicates it is monomeric at temperatures above 13°C. At 4°C, we observed CC1B is dimeric and a complex between CC1B and the IC^1–124^ is formed. Based on this we suspect that the IC binding site lies across the dimeric interface of CC1B and a complex is only able to form when CC1B is a dimer. To test this sedimentation equilibrium experiments were carried out at a fixed speed of 25000 rpm and a scan was taken after 10 and 12 h in 5°C increments from 5 to 25°C with individual protein concentrations of 10 μM. The molecular weight of both CC1B and CC1B + IC^1–124^ decreases with increasing temperatures (Fig. S7A). Similar experiments were performed with CC1. In contrast to CC1B-IC^1–124^ association, there is no change in association of CC1 and IC^1–124^ or CC1 alone with increasing temperatures (Fig. S7B). Taken together, these data indicate that dimer formation of p150^Glued^ is necessary for the p150^Glued^-IC complex to form.

### Dynein-Dynactin Interaction is Governed by Electrostatics

Since the N-terminal region of the dynein IC, IC^1–44^, and the p150^Glued^ CC1 are highly, but oppositely charged at neutral pH (isoelectric points are 9.7 and 4.7, respectively), we hypothesize that the binding is predominantly governed by electrostatic interactions (Fig. S2 panels C and D). To test this, we determined the dissociation constant of the IC-p150^Glued^ interaction as a function of salt and pH.

**Figure 8:**
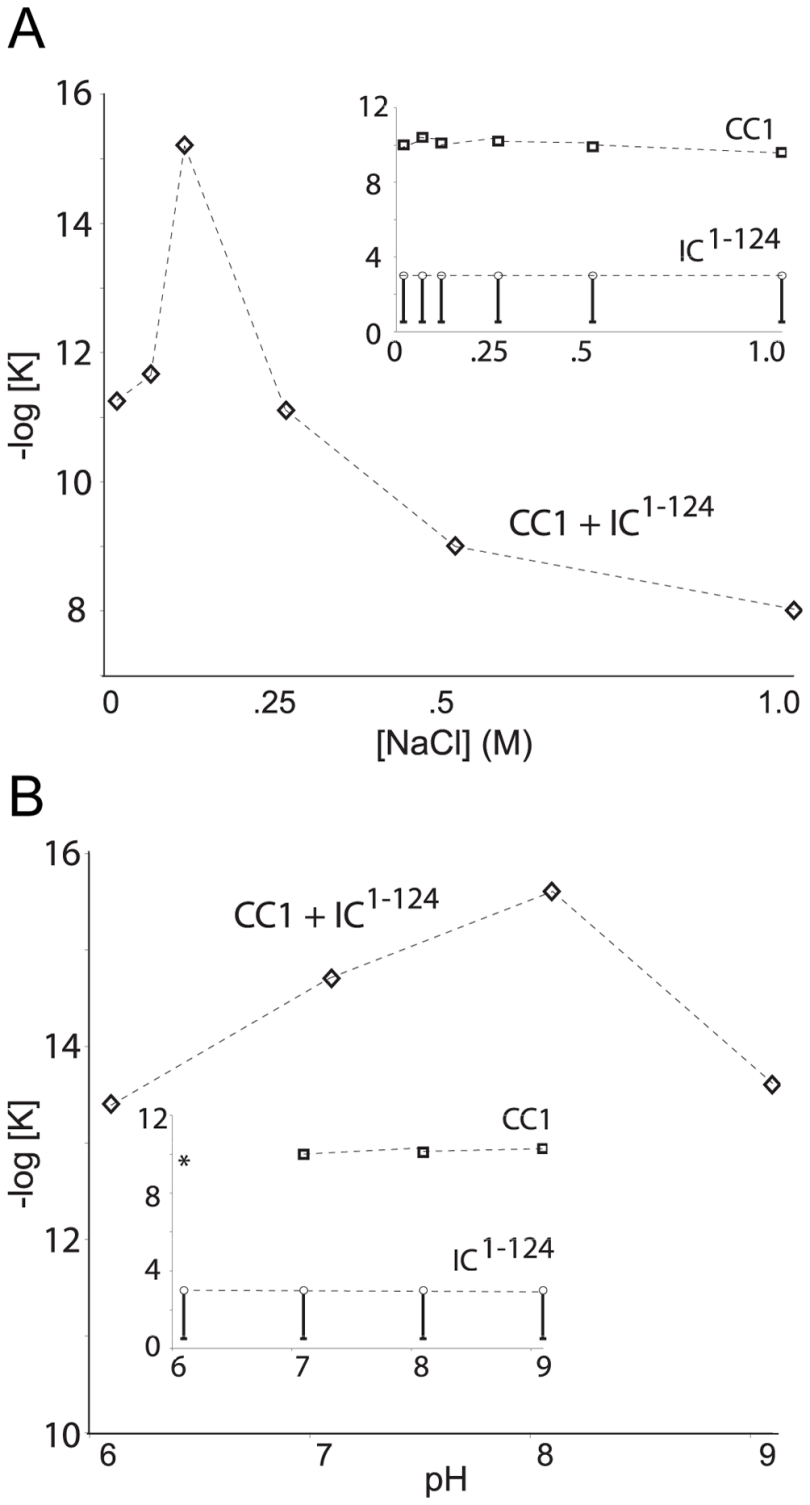
Salt and pH dependence of IC-p150^Glued^ interaction. *A*. Sedimentation equilibrium: SE-AUC of CC1, IC^1–124^ and the CC1-IC^1–124^ complex in the presence of 0, 50, 100, 250, 500 mM and 1.0 M sodium chloride. No change in the oligomeric state of either CC1 or IC^1–124^ occurs with increasing salt (inset). The CC1-IC^1–124^ interaction is strongest at 100 mM sodium chloride and decreases upon increasing salt concentration (fitting analysis is shown in Fig. S8). *B*. SE-AUC of CC1, IC^1–124^ and the CC1-IC^1–124^ complex was run at pH 6.0, 7.0, 8.0 and 9.0. No change in oligomeric state is seen in either CC1 or IC^1–124^. (asterisk denotes that CC1 precipitates at pH 6.0). A strong pH dependence is seen for formation of the CC1-IC^1–124^ complex, where the interaction is the strongest at pH 8.0 and weaker at pH 7.0 and 9.0.

First, we collected sedimentation equilibrium data of IC^1–124^ between 0 and 1 M sodium chloride and observed that this fragment is monomeric to the limits of detection (Fig. 8A inset) Likewise, sedimentation equilibrium experiments of CC1 as a function of salt show no change in hydrodynamic properties between 0 and 1 M sodium chloride. Fitting the sedimentation equilibrium data of CC1 to a monomer-dimer model indicates that the association constant is unchanged as a function of salt. The same set of experiments was performed using a stoichiometric mixture of CC1 and IC1–124. Each complex was fit to a model of CC1_DIMER_ +2 IC fragments → CC1_DIMER_-IC_2_. Other models were explored, but produced worse fits. Plotting the negative log of the calculated dissociation constant using this model indicates a peak at 100 mM sodium chloride. The calculated values for the dissociation constant monotonically decrease with increasing salt concentration above 100 mM (Fig. 8A). Note that the fits are best-fit values. The CC1-IC^1–124^ dissociation constants at 0 and 50 mM NaCl are between 10^−11^ and 10^−13^ M^2^. The CC1-IC^1–124^ dissociation constants at 100, 250, 500 and 1000 mM are ≥10^−15^, 10^−12^, 10^−10^ and 10^−8^ M^2^, respectively. See fitting analysis in Fig. S8.

Next we determined the pH dependence of complex formation using sedimentation equilibrium experiments. First, we observe that CC1 alone is not stable below pH 6.5, however at pH 7.0 and above, it is well-behaved. We observe no significant differences in either CC1 or the IC from pH 7.0 to 9.0 and 6.0 to 9.0, respectively (Fig. 8B, inset). We also observe no difference in the CD spectra of IC ^1–44^ at pH 6.0 or 8.0 (data not shown). The complex was fit to a model of CC1_DIMER_ +2 IC fragments → CC1_DIMER_-IC_2_. The CC1-IC^1–124^ complex contains a maximum in the fitted dissociation constant at pH 8.0, ≤10^−14^ M^2^. Changing the pH of the buffer by one unit results in at least a 10 fold change: with a dissociation constant of ≤10^−13^ M^2^ at pH 7.0 and ≤10^−12^ M^2^ at pH 9.0. Note, in the presence of the IC, CC1 remains in solution. See fitting analysis in Fig. S9.

## Discussion

The dynein-dynactin interaction has been shown to be critical for organelle transport, cytoskeletal organization and cell division [2], [3], [17]. Despite the importance of this interaction, little is known regarding its regulation or energetics. Previous studies have mapped the interaction to the dynein IC and dynactin p150^Glued^ subunits. However, there remains discrepancy in the literature concerning the dynein IC interface on p150^Glued^ [13], [23] as well as the affect of phosphorylation at residue 84 has on dynein-dynactin association [13], [24]. To better understand the interaction as well as to resolve the IC binding site on p150^Glued^, we generated a series of truncation mutants and characterized these mutants by biochemical and biophysical methods. In doing so, we have significantly refined the dynein-dynactin interface. We also provide biochemical evidence that the dynein-dynactin interaction proceeds through a bivalent-bivalent interaction, is predominantly electrostatic, and likely proceeds through a disorder-to-order transition. In addition, we confirmed the mapping results *in vivo* by generating point mutants in the equivalent regions of the yeast IC and quantified their effect on dynein function in cells.

First, we show that the minimal region of the IC that is required for the dynactin interaction spans residues 10 to 44 (in mammals). This mapping is consistent with previous reports where the p150^Glued^ interface was mapped to residues 1 to 106 of the rat IC [11]–[13] and closely matches the sequence identified in recent NMR studies using Drosophila IC [21]. In addition, using the IC truncation constructs, we map the epitope of monoclonal antibodies, mAb70.1 and mAb74.1, to the same region of the IC. Again consistent with our refinement, these antibodies have been demonstrated to block the dynein-dynactin interaction *in vitro*. In addition, these antibodies fail to immunoprecipitate dynactin [11], [14], [45]. Finally, point mutations in this region of Pac11, the yeast IC homolog, significantly affected spindle positioning, similar to loss of either Pac11 or Nip100, the yeast p150^Glued^ homolog [27].

Overall, our interpretation of the free IC data is in agreement with recent NMR studies of the drosophila IC homolog with the exception of the extent of α-helical content, “site 2” [21]. The reason for this discrepancy requires further investigations, but could originate from species differences. Of note, there are six isoforms of cytoplasmic dynein IC in rats, murine and humans. All six differ due to a variable splicing site between the extreme N-terminus (residues 1–44) and the dynein LC binding sites (residues 106–138). While there are multiple isoforms in drosophila dynein IC, each splice site is generated after the dynein LC binding site. Of note, in mammalian dynein, these isoforms are subject to post-translational modifications and thus may have a direct effect on the N-terminus of the IC. For instance, the phosphorylation site mapped to residue 84 in the mammalian IC2C isoform has been implicated in disrupting the dynein-dynactin interaction is distinct from our mapping of the p150^Glued^ binding site on the IC [24], [46]. While this site and others within this disordered region of the mammalian isoforms contain multiple, highly predicted phosphorylation sites (9 sites in IC2C), the sequence that spans the extreme N-terminus and the LC binding site in drosophila only contains one such site (IC S84) and the kinase consensus sequence of this site is not obvious in the rat isoforms. Thus, there are subtle but meaningful differences in the primary sequence that may account for the difference in the biochemical data.

Next, we attempted to resolve the IC binding site on p150^Glued^. While our data strongly indicate that the IC interface requires a dimeric fragment spanning residues 381 to 530, we cannot rule out an additional binding site spanning residues 600 to 811 of p150^Glued^ [23]. Our findings, nonetheless, are consistent with previous studies implicating the dynactin CC1 fragment as the IC binding site [13]. Moreover, overexpression or microinjection of CC1 produces defects in dynein-mediated processes including organelle positioning, implying that CC1 selectively blocks the dynein-dynactin interaction. Finally, we show that forced dimerization of CC1B effectively binds to the dynein IC *in vitro* and *in vivo* and produces similar effects as the expression of the CC1 (Siglin A, Dawn A, Luo Y, Keen J and Williams J, manuscript in preparation). Together, these observations provide substantial evidence that the IC-CC1B interface is required for the dynein-dynactin interaction.

It is also important to note that the interaction shows strong salt and pH dependence. We ascribe this to the highly charged character of each component. Dynein IC^1–44^ is highly, positively charged whereas CC1B is highly, negatively charged. Moreover, we also observe an interaction between dynein IC^1–44^ and the dynactin fragment, CC1A. We currently ascribe this to a non-specific electrostatic interaction, as we did not observe a significant change in the melting temperature, nor a single species in the AUC experiments when CC1A was incubated with IC^1–124^. Future studies will test whether the interaction between dynein IC^1–44^ and dynactin CC1A is physiologically relevant as well as whether the IC N-terminus partitions to negatively charged structures *in vivo*.

Also of note, p150^Glued^ and the C-terminal half of nudE have been reported to bind an overlapping region of the IC N-terminus [14], [15], [47]. Given that the IC-p150^Glued^ interaction is predominantly electrostatic, it is also possible that the nudE binds in a similar manner. We note that the specific region in nudE reported to bind to the dynein IC, residues 256–291, has an isoelectric point of 9.2, not 4.4 as observed for CC1B. Moreover, this region has been shown to be intrinsically disordered [15]. These observations suggest that p150^Glued^ and nudE bind to the IC by different mechanisms.

Based on this mapping and results in the literature, CC1B is predicted to be at the ‘shoulder’ of the dynactin complex as observed in electron microscopy images [43]. This is also the same region in the dynactin complex that dynactin subunits, p50 and p24, are proposed to bind p150^Glued^ [48]. Similar to *in vivo* studies using CC1, the overexpression of p50 is also implicated in blocking the dynein-dynactin interaction, albeit by a completely different mechanism (e.g., p50 is proposed to disrupt the Arp rod from dynactin) [49]. In addition, the secondary structure, sedimentation equilibrium results and chemical cross linking data suggest that the p150^Glued^ CC1A fragment comprises the ‘arm’ observed in the EM micrographs [43]. Deletion of the equivalent region in Nip100, the yeast homolog of p150^Glued^, does not affect spindle positioning though it does affect the dynein processivity or run length [50]. Taken together, the proximity of proposed position of the refined IC binding site (e.g., CC1B) and p50 and p24 within the dynactin complex and their effect of the overexpression/microinjection on dynein-mediated processes *in vivo* suggests that the IC^1–44^ and CC1B is the primary interface governing the dynein-dynactin interaction.

Characterization of the hydrodynamic properties of these fragments also suggests a potential mechanism that governs the dynein-dynactin interaction. Specifically, sedimentation equilibrium of the IC^1–44^ only (as well as IC^1–124^ only) shows that the N-terminus is monomeric under all conditions. This is also consistent with the lack of secondary structure observed by CD and no thermal transition. In the context of the dynein complex, the C-terminal WD domains of the dynein IC bind to the dimeric heavy chain [51]. In addition, the dimeric dynein LCs, TcTex1, LC8 and LC7, bind to the IC at residues 112–125, 129–137, and 180–280, respectively, and further enforce the dimerization of the IC [25], [52]. On the other hand, there are no further restraints on the highly positively charged, N-terminal 44 residues. Thus, we argue that this region remains disordered in the dynein complex, absent dynactin.

The IC binding site of p150^Glued^, on the other hand, is dimeric in the context of the dynactin complex. Specifically, the sedimentation equilibrium data show that the CC1B fragment of p150^Glued^ is a dimer at temperatures below 13°C. Moreover, CC1A, which immediately precedes CC1B, is also dimeric. Finally, a second coiled-coil region in p150^Glued^, CC2 (residues 928–1049), is also dimeric (A Siglin & J Williams, unpublished observation). Additionally, we observe a substantially higher melting point for CC1 compared to CC1A or CC1B, suggesting favorable multivalent interactions. Thus, in the context of the p150^Glued^ and the dynactin complex, CC1B is embedded between two homodimeric coiled-coils of p150^Glued^ that strongly favors its dimerization.

Based on these observations, we suggest that CC1B acts as a folded template for the dynein IC^1–44^ to bind and potentially ‘folds’ on to, akin to a disordered-to-ordered transition found in many other systems including the well studied KIX/CREB interaction [53]. More importantly, the hydrodynamic data indicate that the interaction proceeds through a “dimer of dimers”. While not entirely unexpected [25], there are a number of important ramifications in these experimental observations. First, interactions involving disordered regions are generally weak (e.g., entropic penalty upon binding). By turning to a bivalent-bivalent interaction, significant gains in apparent affinity are frequently observed [54], [55]. We note that our measured dissociation constant range for this interaction by AUC is 10^−11^ to 10^−13^ M^2^. This is close in line with the isothermal titration measurements with the Drosophila IC and p150^Glued^ CC1 fragment, which yielded values of K_D_  = 3.5 μM [21]. Physically tethering the ICs on the other hand could decrease the binding concentration affinity of the full dynein complex to the full dynactin complex to picomolar levels [54], [55]. Presumably, such a high affinity interaction is potentially necessary as a strong interaction would be necessary to transport vesicles (e.g., assuming a diffusion limited on-rate, k_ON_ = 10^8^ M^−1^s^−1^ and a K_D_  = 3.5 μM for the monomeric interaction, the life time of the complex would be ∼2 ms). Second, a bivalent-bivalent interaction effectively enhances the specificity of the interaction. Specifically, this arrangement requires two binding sites of the receptor to be optimally spaced and oriented to optimally interact with the bivalent ligand. In fact, earlier on in this study, we recognized that either the IC or p150^Glued^ or both must be monomeric prior to the interaction. This realization is distinct from previous assumptions that the N-terminus of the dynein IC is a preformed coiled-coil and that this coiled-coil binds to a coiled-coil fragment of the dynactin p150^Glued^ [2]. Such an interaction of two preformed coiled-coils would produce an asymmetric structure and potentially lead to aggregation. We note that in sedimentation equilibrium experiments we observe the hetero-tetramer complex and did not find evidence of higher molecular weight species consistent with an oligomer. Finally, a bivalent-bivalent interaction permits rapid regulation by post-translation modification. Specifically, due to the weak affinity of each half-site, post-translational modification of one half of the dimer (e.g., one IC chain) that affects it's binding (directly or through a conformational change) will lead to the rapid dissociation of the complex. Taken together, the dimeric organization of the dynein complex and the p150^Glued^ confers a number of advantages in forming and regulating this interaction. We note that other proteins that are well characterized and bind to the dynein IC are also dimeric (e.g., Lis1, Nde1) and suspect that their bivalent character is critical to their function in regulating dynein activity.

**Figure 9:**
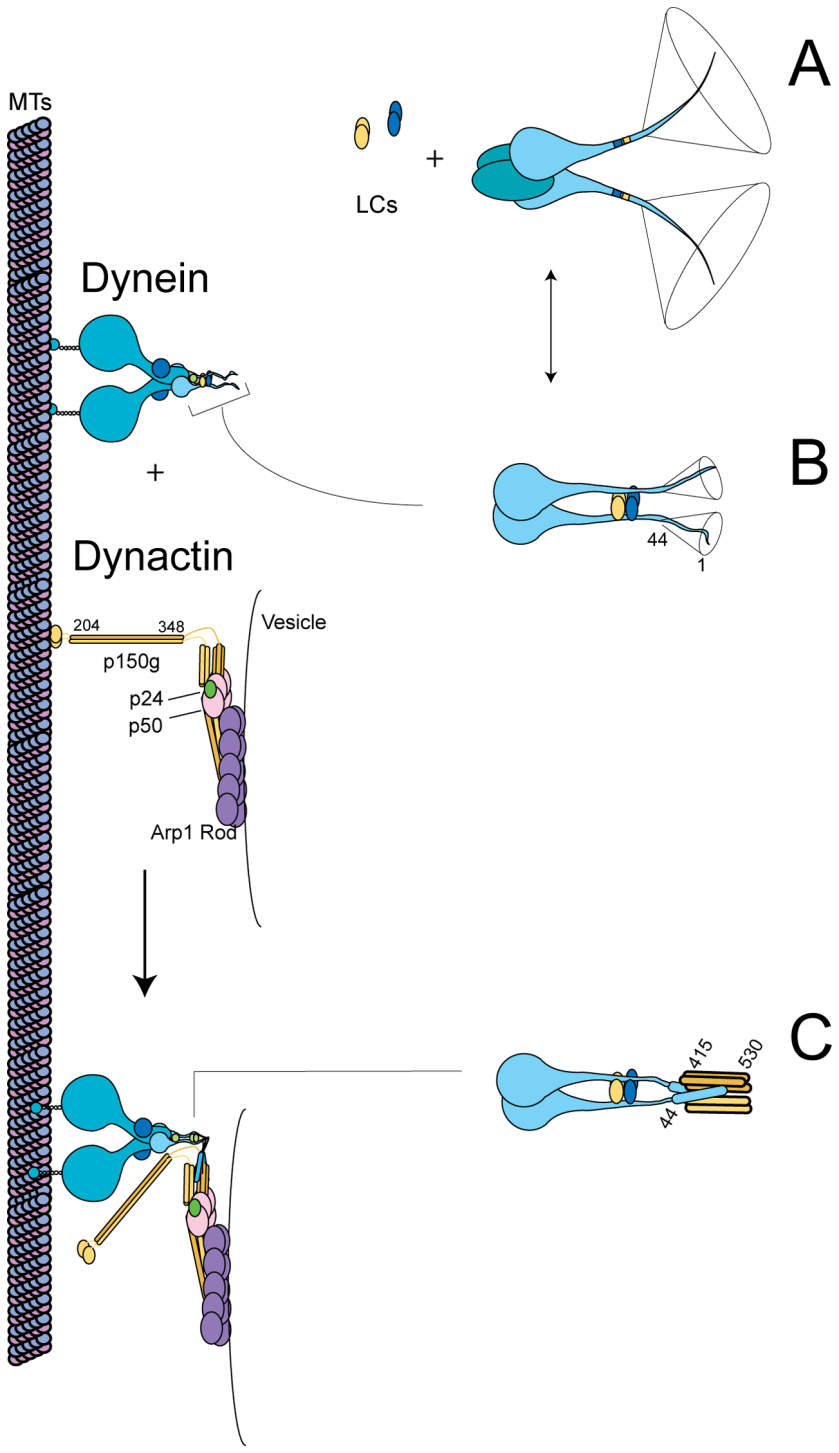
Proposed mechanism of dynein-dynactin binding. Based on the refinement of the dynein-dynactin interaction and the physical characterization of the individual fragments, we propose that the N-terminus of the dynein IC is disordered in the in the absence of dynactin. Further, based on recent data from our lab and others we propose that the dynein LCs affect the N-termini by reducing and/or orienting the N-termini of the IC to optimally bind to dynactin. Taken together, we suggest the following model: *A*. The N-terminus of the intermediate chain exists in a disordered state. *B*. Upon IC binding to the light chains the radius of gyration of the disordered region is reduced. *C*. The N-termini of the IC bind to p150^Glued^ CC1B located in the shoulder.

In addition, our recent structural and cell-based assays investigating the role of the dynein LCs on dynein function suggest that the LCs regulate dynein function [26], [27]. The dynein LCs bind to the dynein IC immediately C-terminal to the dynactin binding site. Specifically, we observe that sequestering the dynein LCs, LC8 and TcTex1, using an inducible molecular trap rapidly affects endosome and lysosome positioning [26], [27]. Based on the ‘monomeric’ character of the IC N-terminus, we propose that the presence of the LCs is necessary to orient and reduce the radius of gyration of the ICs (Fig. 9) and strengthening the dynein-dynactin interaction.

In summary, the extensive biochemical and biophysical characterization of the IC-p150^Glued^ interaction presented here reveals that the interaction precedes through a bivalent-bivalent interface, is governed by electrostatic interactions and undergoes a disorder-to-order transition.

## Supporting Information

Figure S1 **Alignment of dynein IC and p150^Glued^ sequences from different organisms and predicted coiled-coil regions.**
(TIF)

Figure S2 **Coiled-coil and isoelectric point sequence analysis of IC and p150^Glued^ binding regions.** (A/B) Both the intermediate chain and p150^Glued^ contain regions that have a propensity to exist in a coiled-coil, denoted by a P-score of 0.025 or less (1). Coiled-coil prediction programs indicate a possible N-terminal coiled-coil spanning residues 1–44 of the IC (A). In addition, sequence alignment (2) and coiled-coil prediction of p150^Glued^ indicate a conserved break in the coiled-coil region. Based on this break we designed two new fragments denoted CC1A and CC1B (B). (C/D) The average isoelectric point of the intermediate chain, residues 1–151 and p150^Glued^ CC1 was determined by calculating the isoelectric point for 28 residues, every 7 residues. Both the p150^Glued^ and IC binding sites are highlighted with a grey box. Note that the isoelectric point of the IC^1–44^ is 9.7, while p150^Glued^ (415–530) is 4.43, indicating the interaction may primarily be governed by electrostatic interactions.
(TIF)

Figure S3 **Alanine scanning mutagenesis of Pac11 and statistical analysis of spindle position assays**. (A) Native PAGE indicates that points mutants Pac11-L4A,K5A,Q6A, Pac11-Q6A,L7A,E8A, Pac11-E9A,K10A,R11A,Pac11-L17A,R18A, and Pac11-E19A,R20A,R21A abrogate Pac11-p150Glued CC1B binding. In the presence of LC8, Pac11-p150^Glued^ CC1B binding is restored (indicated by an asterisk). Note only a slight change in migration is seen for the Pac11-p150^Glued^-LC8 complexes, however the CC1B band is absent or reduced indicating incorporation into the complex (arrow). Figure is composed of four separate native PAGE gels. (B) P-values were determined by t-test for mitotic spindle position assay.
(TIF)

Figure S4 **Chi square analysis of p150^Glued^ oligomer formation and IC-p150^Glued^ complex formation.** (A) The radial absorbance of the CC1, CC1A and CC1B constructs was fit to a monomer-dimer model affording an optimal dissociation constant. Next, the dissociation constant was fixed at different values centered about the best-fit value and the resulting chi squared value was determined. The chi squared was plotted against the fixed dissociation constants. A sharp rise in the chi squared value indicates limiting values. For instance, the lower limit of the dissociation constant is 8 for CC1(A, left panel). However, the value may be much greater. (B) The radial absorbance of IC^1–124^ mixed with CC1, CC1A and CC1B was fit to a 2IC +CC ⇔ (IC)_2_(CC)_1_ model affording an optimal dissociation constant. The same chi square analysis was perfomed. Note that CC1B + IC^1–124^ is well constrained at 12. (B) The IC-p150^Glued^ complex data was fit by fixing the dissociation constant around the best-fit value and the chi squared value was recorded.
(TIF)

Figure S5 **p150^Glued^ CC1 is a parallel coiled-coil.** (A) Upon incubation of Cys-CC1 with 3,5-DMBA we see the presence of a band equivalent to two times the molecular weight of Cys-CC1 (arrow). This indicates that the two cysteines are in close proximity in the CC1 dimer and are able to be crosslinked.
(TIF)

Figure S6 **Thermal Denaturation of p150^Glued^ fragments with IC^1–44^.** (A) CC1A alone (diamonds) or incubated with an equimolar concentration of IC^1–44^ (squares). (B) CC1B alone (diamonds) or incubated with an equimolar concentration of IC^1–44^ (squares).
(TIF)

Figure S7 **Dimerization and temperature dependence of IC-p150^Glued^ interaction.** (A/B) Oligomeric state and association with IC^1–124^ was examined as a function of temperature for both CC1B and CC1. A single speed of 25000 rpm was analyzed at 5, 10, 15, 20 and 25°C. The dimerization of CC1B and association with IC^1–124^ is temperature dependent (A), while no change in either dimerization or association is seen for CC1 (B). IC^1–124^ is monomeric at all temperatures.
(TIF)

Figure S8 **Chi square analysis of p150^Glued^ oligomer (A) and IC-p150^Glued^ complex (B) in salt dependence assays.**
(TIF)

Figure S9 **Chi square analysis of p150^Glued^ oligomer (A) and IC-p150^Glued^ complex (B) in pH dependence assays.**
(TIF)
