# Correction: Dynein and Dynactin Leverage Their Bivalent Character to Form a High-Affinity Interaction

**DOI:** 10.1371/journal.pone.0304916

**Published:** 2024-06-04

**Authors:** Amanda E. Siglin, Shangjin Sun, Jeffrey K. Moore, Sarah Tan, Martin Poenie, James D. Lear, Tatyana Polenova, John A. Cooper, John C. Williams

This correction addresses errors in Figs 2, 3 and S3 of [1].

**Fig 2:**
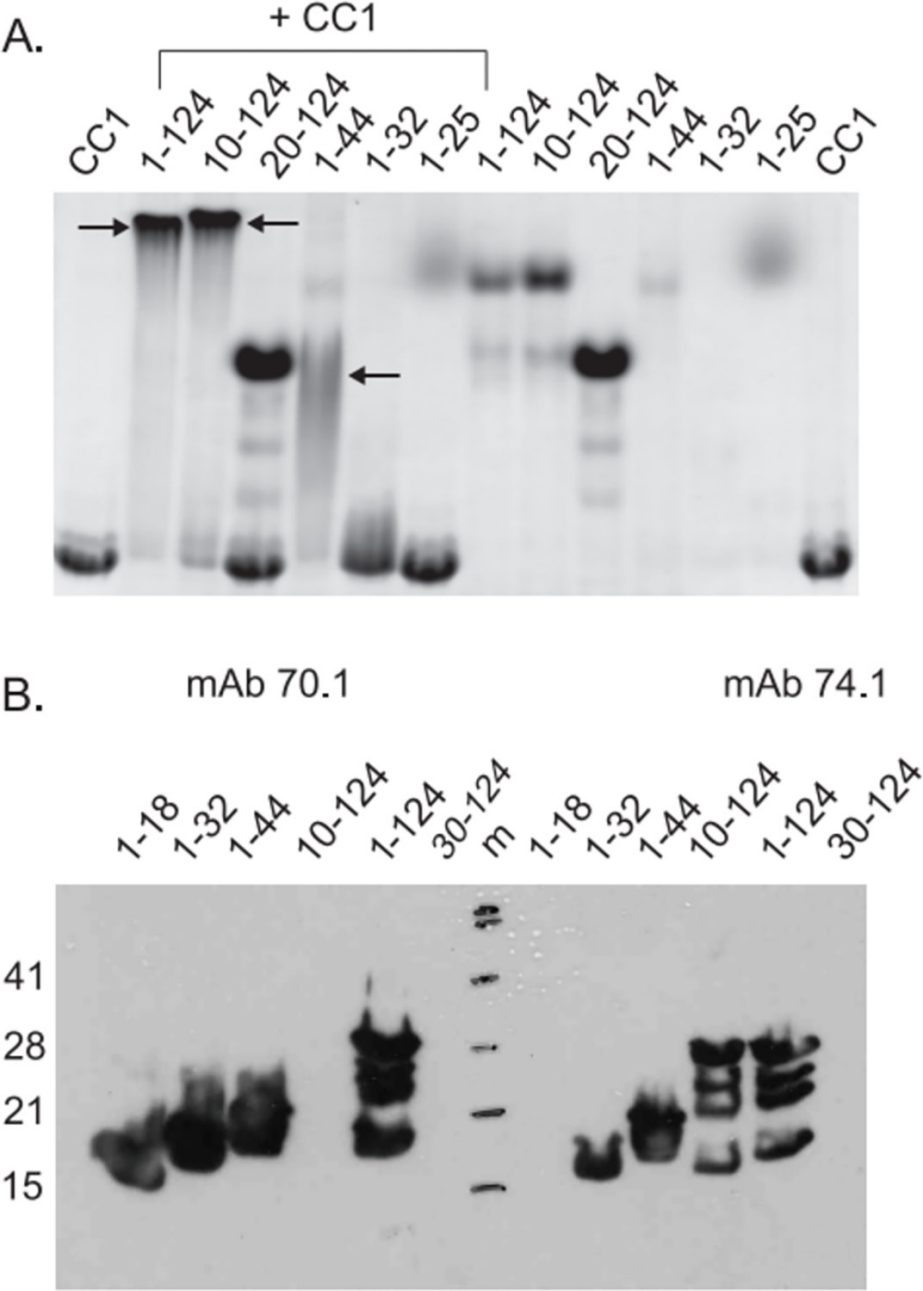
Dynein intermediate chain minimal binding domain. *A*. Native PAGE indicates that residues 10–44 are sufficient for binding to p150^Glued^ CC1. A gel shift indicates IC2C fragments spanning residues 1–124, 10–124 and 1–44 are capable of binding CC1 (indicated by arrows). However, fragments spanning 20–124 and 1–32 are not able to bind to CC1. It is important to note that due to the large negative charge of the IC some constructs do not enter the gel. *B*. Epitope mapping of IC antibodies: The epitopes of α-IC mAb 70.1 and 74.1 are located within the p150^Glued^ binding domain. Specifically, α-IC 70.1 recognizes the region between residues 1–18 and α-IC 74.1 recognizes 10–30. https://doi.org/10.1371/journal.pone.0059453.g002.

**Fig 3:**
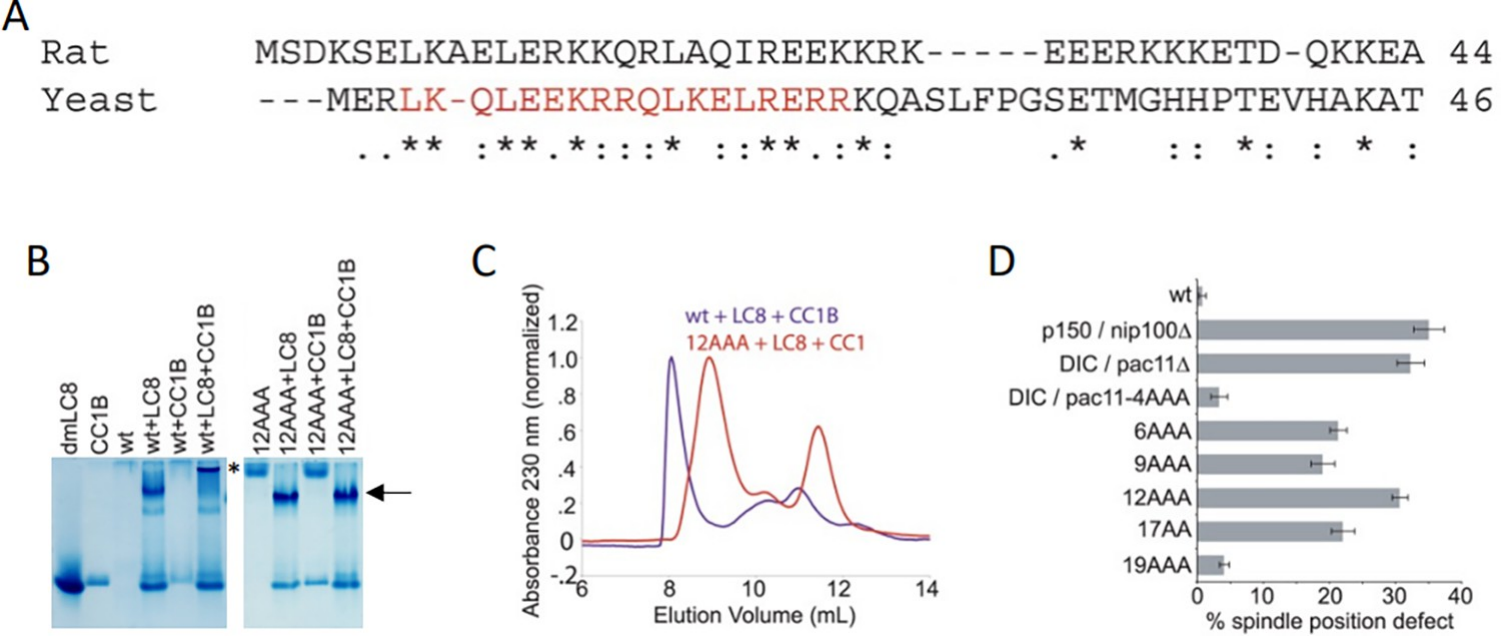
Alanine scanning mutagenesis of Pac11. *A*. Sequence alignment of *R*. *norvegicus* IC and *S*. *cerevisiae* Pac11 (IC). Alanine point mutations were introduced into Pac11 (indicated in red). A (*) indicates identical amino acids, (:) indicates highly conserved, similar amino acids, (.) indicates amino acids that are somewhat similar and blank indicates dissimilar amino acids. *B*. Native PAGE indicates that *S*. *cerevisiae* wt-Pac11^1–86^ is capable of binding p150^Glued^ CC1B alone (indicated by the loss of CC1B) and in the presence of dynein light chain 8 (LC8) (indicated by the gel shift of LC8). Due to the charge and hydrodynamic properties of wt-Pac11^1–86^, it does not enter the native PAGE. An asterisk indicates gel shift upon wt- Pac11^1–86^-LC8-CC1B binding. Pac11^1–86^ triple point mutant Pac11-R12A,Q13A,L14A (12AAA) is unable to bind p150^Glued^ CC1B alone or in the presence of LC8. Arrow indicates formation of Pac11-R12A,Q13A,L14A-LC8 (12AAA) binding. No gel shift occurs upon addition of CC1B. *C*. Size exclusion chromatography indicates wt-Pac11^1–86^ forms a complex with LC8 and CC1B (purple), with an elution volume of 8.07 mL, while Pac11- R12A,Q13A,L14A (12AAA) is unable to bind to CC1B (red). The Pac11-R12A,Q13A,L14A(12AAA)-LC8 complex elutes at 8.97 mL and CC1B elutes at 11.45 mL. *D*. Spindle positioning in wild-type and mutant cells expressing GFP-labeled microtubules. The percentage of cells exhibiting spindle position defects (see Materials and Methods) was determined for wild-type (yJC5919), p150^Glued^/*nip100*∆ (yJC6047), DIC/*pac11*∆ (yJC6354), DIC/*pac11*-4A (L4A,K5A,Q6A, yJC6916), *pac11*-6A (Q6A,L7A,E8A, yJC6917), *pac11*-9A (E9A,K10A,R11A, yJC6918), *pac11*-12A (R12A,Q13A,L14A, yJC6846), *pac11*-17A (L17A,R18A, yJC6847), and *pac11*-19A (E19A,R20A,R21A, yJC6919) strains. Error bars denote SEM. P-values are shown in S3B Fig. https://doi.org/10.1371/journal.pone.0059453.g003.

Specifically:

- In the originally published Fig 2B, the mAb 74.1 30–124 lane is incorrect and is a duplicate of the mAb 74.1 1–18 lane when rotated 180 degrees.
- In the originally published Fig 3D, there is an undeclared splice line between lanes 6 and 7.
- In the originally published S3B Fig, there are undeclared splice lines between lanes 2 and 3, 6 and 7, 10 and 11, 14 and 15, and 18 and 19.
- The originally published Fig 3B and 3D are swapped in error and do not match the Fig 3 figure legend.
- The originally published S3A and S3B Fig are swapped in error and do not match the S3 Fig figure legend.

Regarding Fig 3D and S3B Fig in [1], the corresponding author stated that both figures were prepared from multiple gels as the full experiments did not fit on a single gel. These gels were then combined to produce Fig 3D and S3B Fig in [1].

Updated versions of Figs 2, 3, and S3 are provided with this Correction to address the above issues. The revised Fig 2B includes the correct original gel. Please note the updated Fig 2A and the corresponding labels have been inverted compared to the originally published Fig 2A in [1]. In the updated Fig 3 and S3 Fig, spaces have been added between lanes from distinct gels. Fig 3B and 3D and S3A and S3B Fig have been updated to match the figure legends for Fig 3 and S3 Fig.

The original uncropped images underlying the updated Figs 2, 3, and S3 are available in S1–8 Files provided with this notice.

The corresponding author stated that the individual-level quantitative data underlying this article [1] are no longer available.

The authors acknowledge, apologize, and are grateful for the opportunity to correct unintentional errors in the published article.

## Supporting information

S3 Fig Alanine scanning mutagenesis of Pac11 and statistical analysis of spindle position assays. (A) Native PAGE indicates that points mutants Pac11-L4A,K5A,Q6A, Pac11-Q6A,L7A,E8A, Pac11-E9A,K10A,R11A,Pac11-L17A,R18A, and Pac11-E19A,R20A,R21A abrogate Pac11-p150^Glued^ CC1B binding. In the presence of LC8, Pac11-p150^Glued^ CC1B binding is restored (indicated by an asterisk). Note only a slight change in migration is seen for the Pac11-p150^Glued^-LC8 complexes, however the CC1B band is absent or reduced indicating incorporation into the complex (arrow). Fig is composed of four separate native PAGE gels. (B) P-values were determined by t-test for mitotic spindle position assay. https://doi.org/10.1371/journal.pone.0059453.s003.
(PDF)

S1 File Original underlying image of labeled gel presented in Fig 2B. This file includes the original uncropped image used for Fig 2B on slide 1, and a repeat experiment, conducted at a later date, on slide 2.
(PPTX)

S2 File Original underlying images of labeled gels represented in Fig 3B and S3A Fig. This file includes the two original uncropped images used to compose the updated Fig 3B on slide 1, and the four original uncropped images used to compose the updated S3A Fig on slides 2 and 3.
(PPTX)

S3 File Original underlying image for gel presented in Fig 2B. (JPG)

S4 File Original underlying image for gel presented in Fig 3B and S3A Fig. This file contains the original uncropped image for which lanes 3–6 correspond to lanes 7–10 of Fig 3B, and lanes 7–10 correspond to lanes 15–18 of S3A Fig.
(PDF)

S5 File Original underlying image for gel presented in Fig 3B and S3A Fig. This file contains the original uncropped image for which lanes 1–6 correspond to lanes 1–6 of Fig 3B and lanes 7–10 correspond to lanes 3–6 of S3A Fig.
(PDF)

S6 File Original underlying image for gel used in repeat experiment for data shown in Fig 3B and S3A Fig. This file contains the original uncropped image for a repeat experiment conducted at an earlier date. Lanes 1–6 are a repeat experiment of lanes 1–6 in Fig 3B and lanes 7–10 are a repeat experiment of lanes 3–6 in S3A Fig.
(PDF)

S7 File Original underlying image for gel presented in S3A Fig. This file contains the original uncropped image for which lanes 3–10 correspond to lanes 7–14 of S3A Fig.
(PDF)

S8 File Original underlying image for gel presented in S3A Fig. This file contains the original uncropped image for which lanes 1–10 correspond to lanes 1, 2 and 19–26 of S3A Fig.
(PDF)
