# Supplementary figures and images for: Dynein and Dynactin Leverage Their Bivalent Character to Form a High-Affinity Interaction
Source: PLoS One. 2013 Apr 5;8(4):e59453. doi: 10.1371/journal.pone.0059453 (PMC3618186; doi:10.1371/journal.pone.0059453)

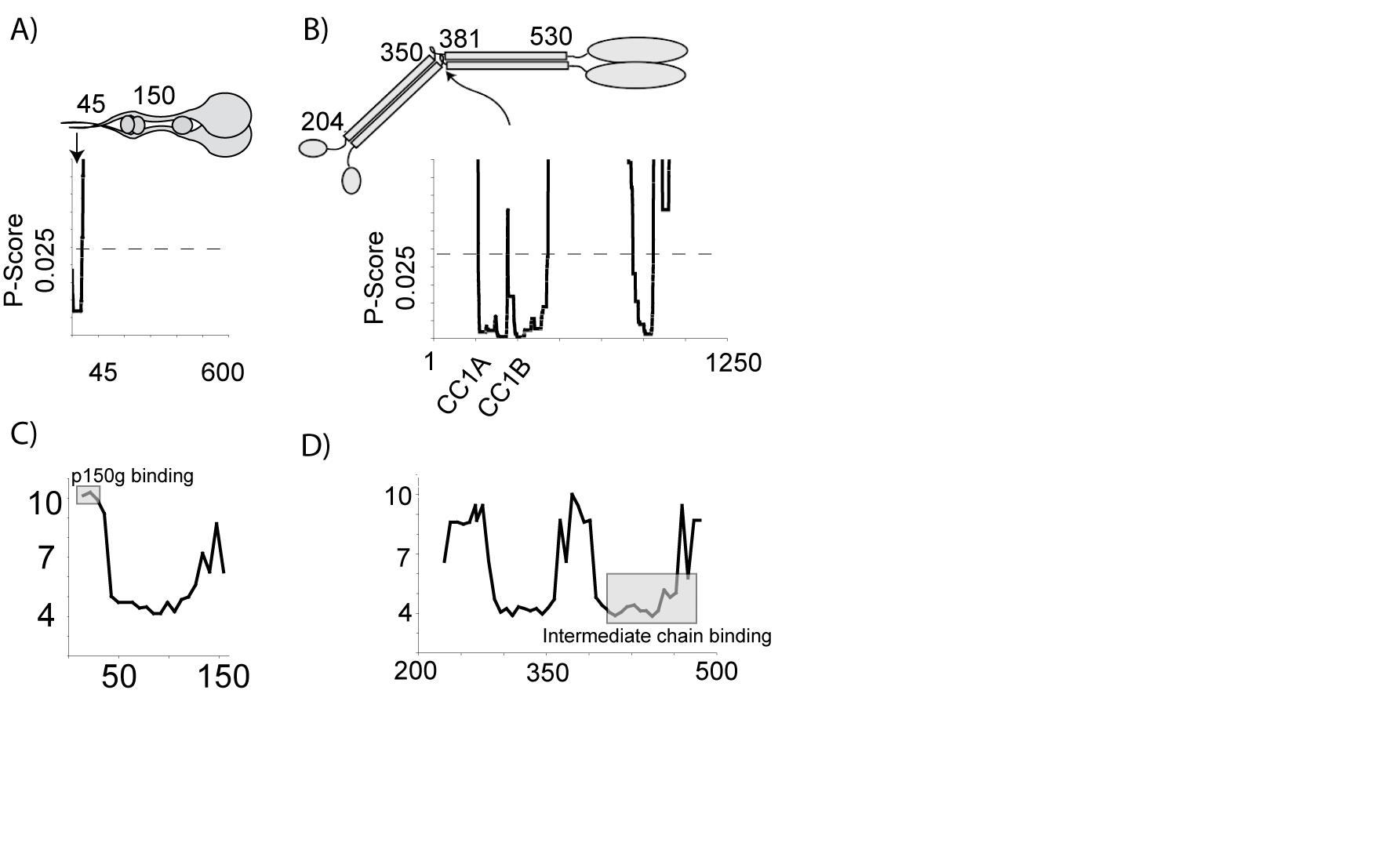

Supplement: Figure S1 — Alignment of dynein IC and p150Glued sequences from different organisms and predicted coiled-coil regions. (TIF) [file pone.0059453.s001.tif]

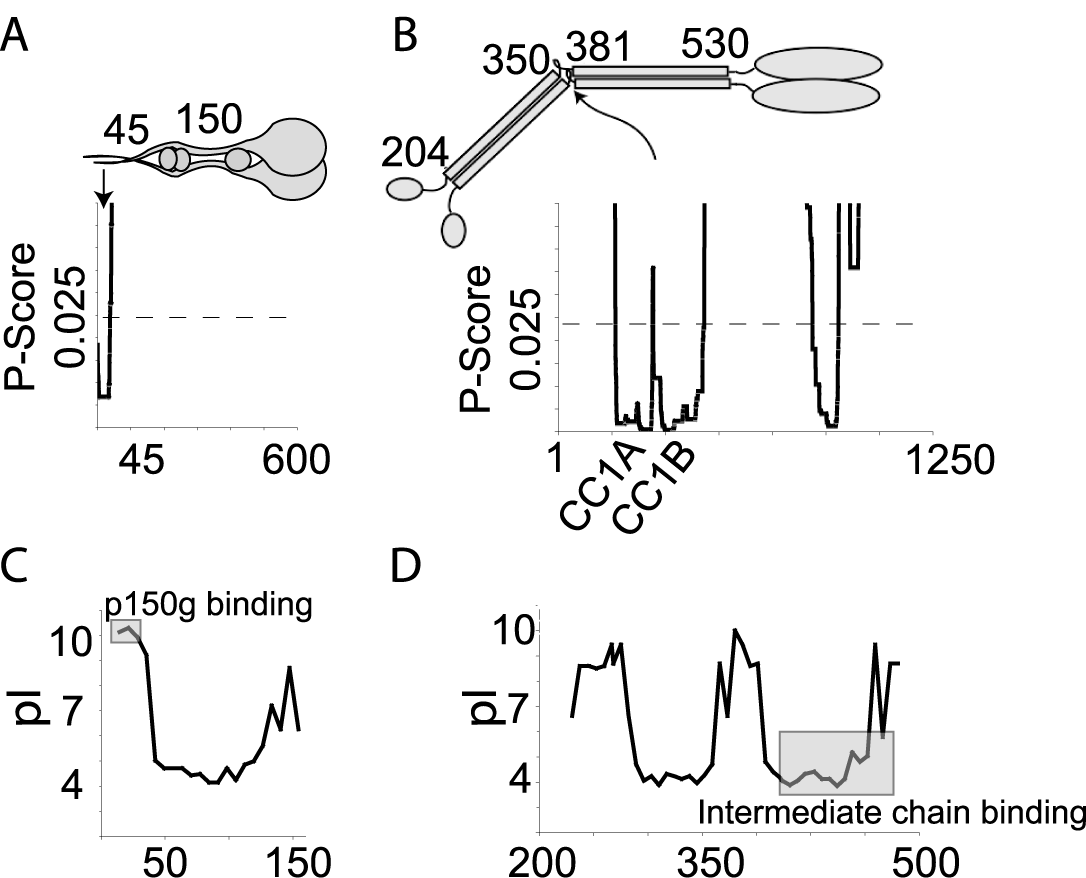

Supplement: Figure S2 — Coiled-coil and isoelectric point sequence analysis of IC and p150Glued binding regions. (A/B) Both the intermediate chain and p150Glued contain regions that have a propensity to exist in a coiled-coil, denoted by a P-score of 0.025 or less (1). Coiled-coil prediction programs indicate a possible N-terminal coiled-coil spanning residues 1–44 of the IC (A). In addition, sequence alignment (2) and coiled-coil prediction of p150Glued indicate a conserved break in the coiled-coil region. Based on this break we designed two new fragments denoted CC1A and CC1B (B). (C/D) The average isoelectric point of the intermediate chain, residues 1–151 and p150Glued CC1 was determined by calculating the isoelectric point for 28 residues, every 7 residues. Both the p150Glued and IC binding sites are highlighted with a grey box. Note that the isoelectric point of the IC1–44 is 9.7, while p150Glued (415–530) is 4.43, indicating the interaction may primarily be governed by electrostatic interactions. (TIF) [file pone.0059453.s002.tif]

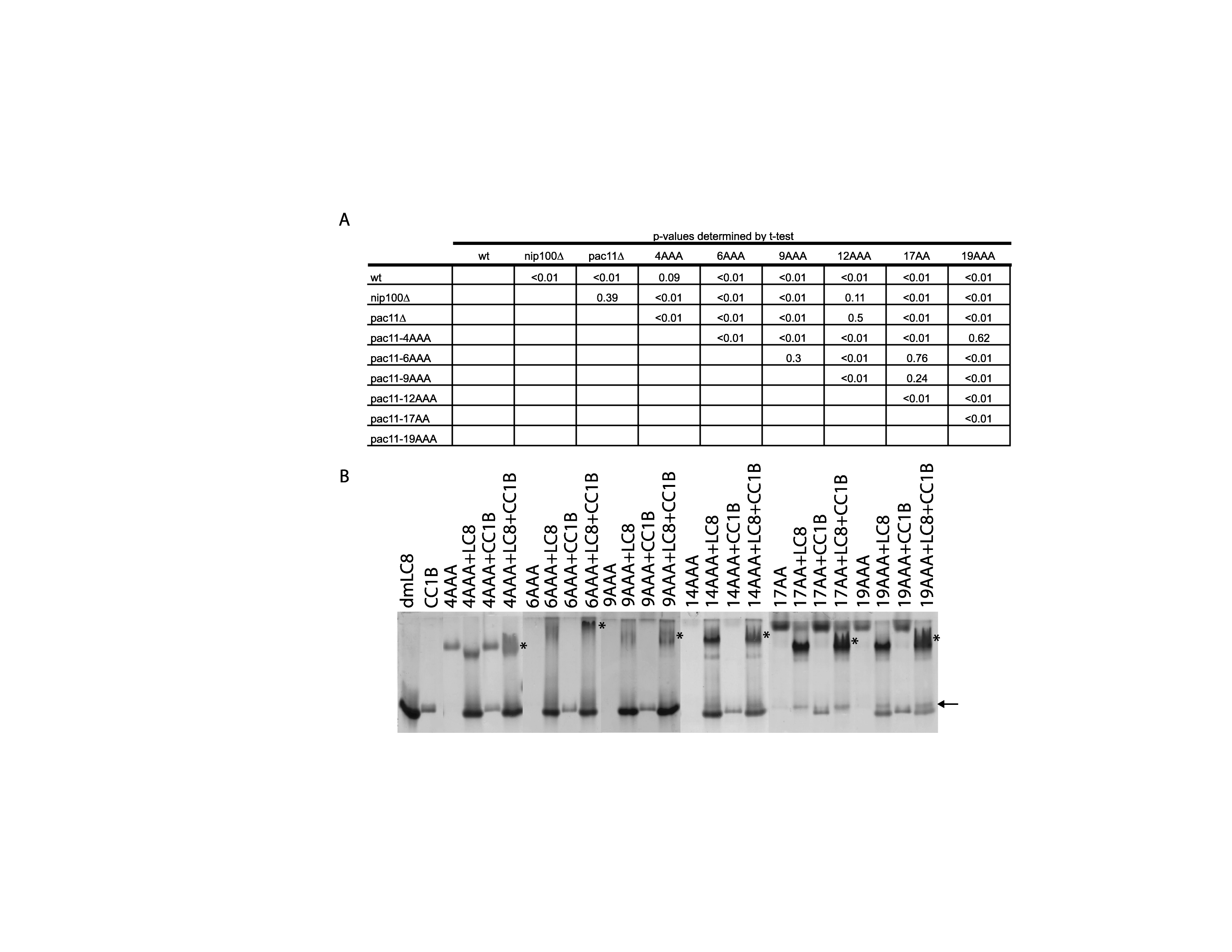

Supplement: Figure S3 — Alanine scanning mutagenesis of Pac11 and statistical analysis of spindle position assays. (A) Native PAGE indicates that points mutants Pac11-L4A,K5A,Q6A, Pac11-Q6A,L7A,E8A, Pac11-E9A,K10A,R11A,Pac11-L17A,R18A, and Pac11-E19A,R20A,R21A abrogate Pac11-p150Glued CC1B binding. In the presence of LC8, Pac11-p150Glued CC1B binding is restored (indicated by an asterisk). Note only a slight change in migration is seen for the Pac11-p150Glued-LC8 complexes, however the CC1B band is absent or reduced indicating incorporation into the complex (arrow). Figure is composed of four separate native PAGE gels. (B) P-values were determined by t-test for mitotic spindle position assay. (TIF) [file pone.0059453.s003.tif]

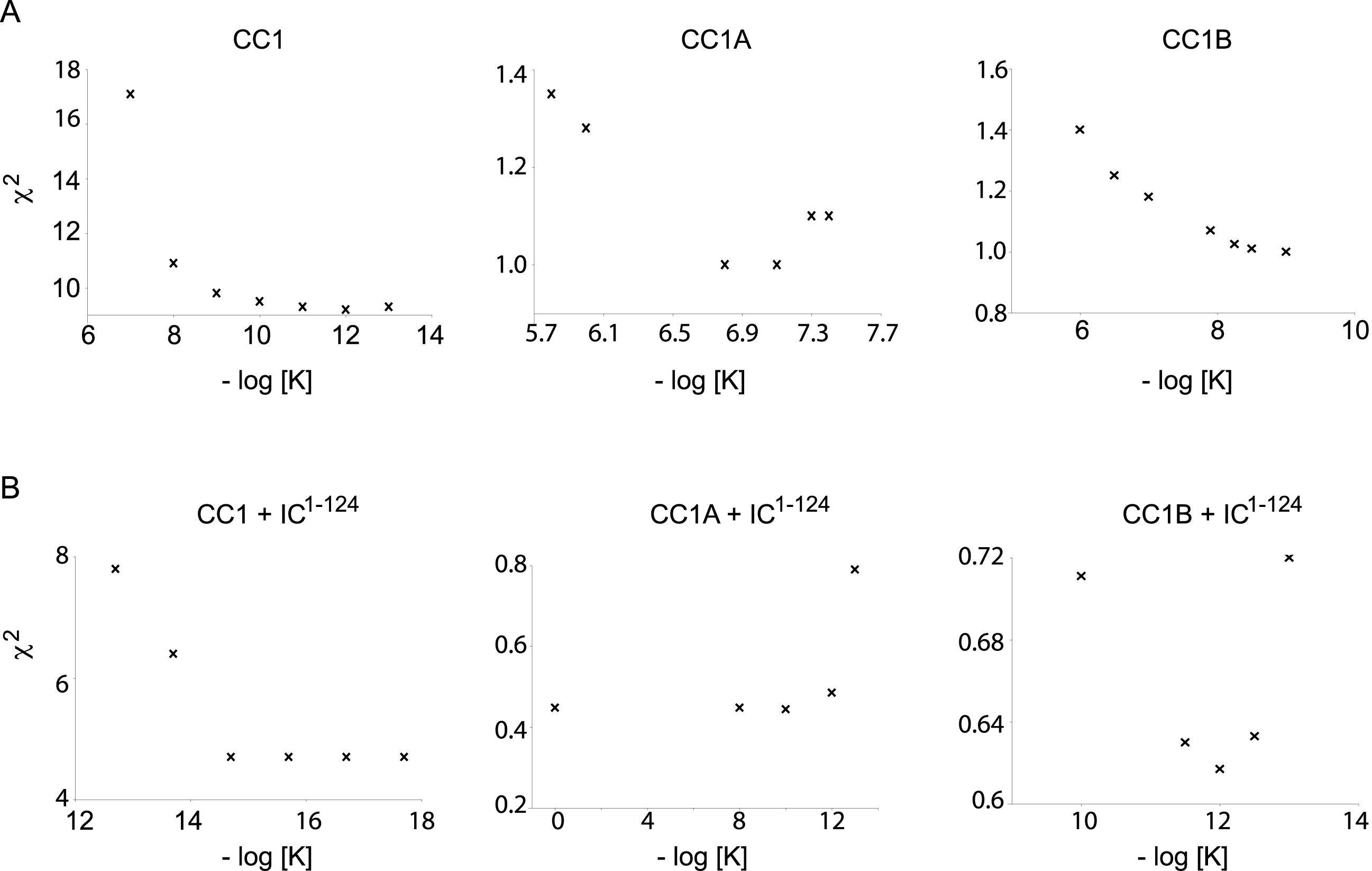

Supplement: Figure S4 — Chi square analysis of p150Glued oligomer formation and IC-p150Glued complex formation. (A) The radial absorbance of the CC1, CC1A and CC1B constructs was fit to a monomer-dimer model affording an optimal dissociation constant. Next, the dissociation constant was fixed at different values centered about the best-fit value and the resulting chi squared value was determined. The chi squared was plotted against the fixed dissociation constants. A sharp rise in the chi squared value indicates limiting values. For instance, the lower limit of the dissociation constant is 8 for CC1(A, left panel). However, the value may be much greater. (B) The radial absorbance of IC1–124 mixed with CC1, CC1A and CC1B was fit to a 2IC +CC ⇔ (IC)2(CC)1 model affording an optimal dissociation constant. The same chi square analysis was perfomed. Note that CC1B + IC1–124 is well constrained at 12. (B) The IC-p150Glued complex data was fit by fixing the dissociation constant around the best-fit value and the chi squared value was recorded. (TIF) [file pone.0059453.s004.tif]

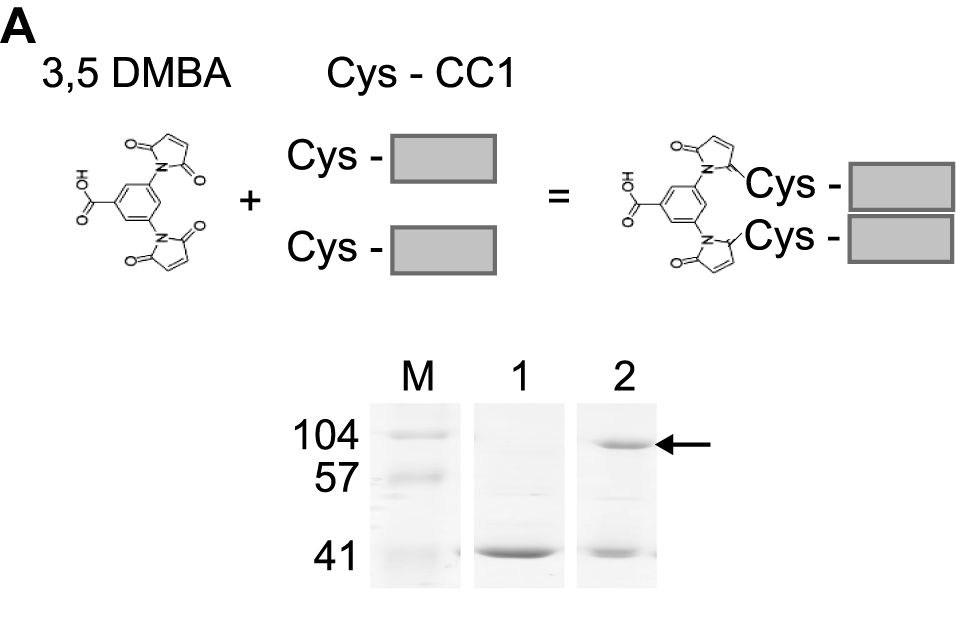

Supplement: Figure S5 — p150Glued CC1 is a parallel coiled-coil. (A) Upon incubation of Cys-CC1 with 3,5-DMBA we see the presence of a band equivalent to two times the molecular weight of Cys-CC1 (arrow). This indicates that the two cysteines are in close proximity in the CC1 dimer and are able to be crosslinked. (TIF) [file pone.0059453.s005.tif]

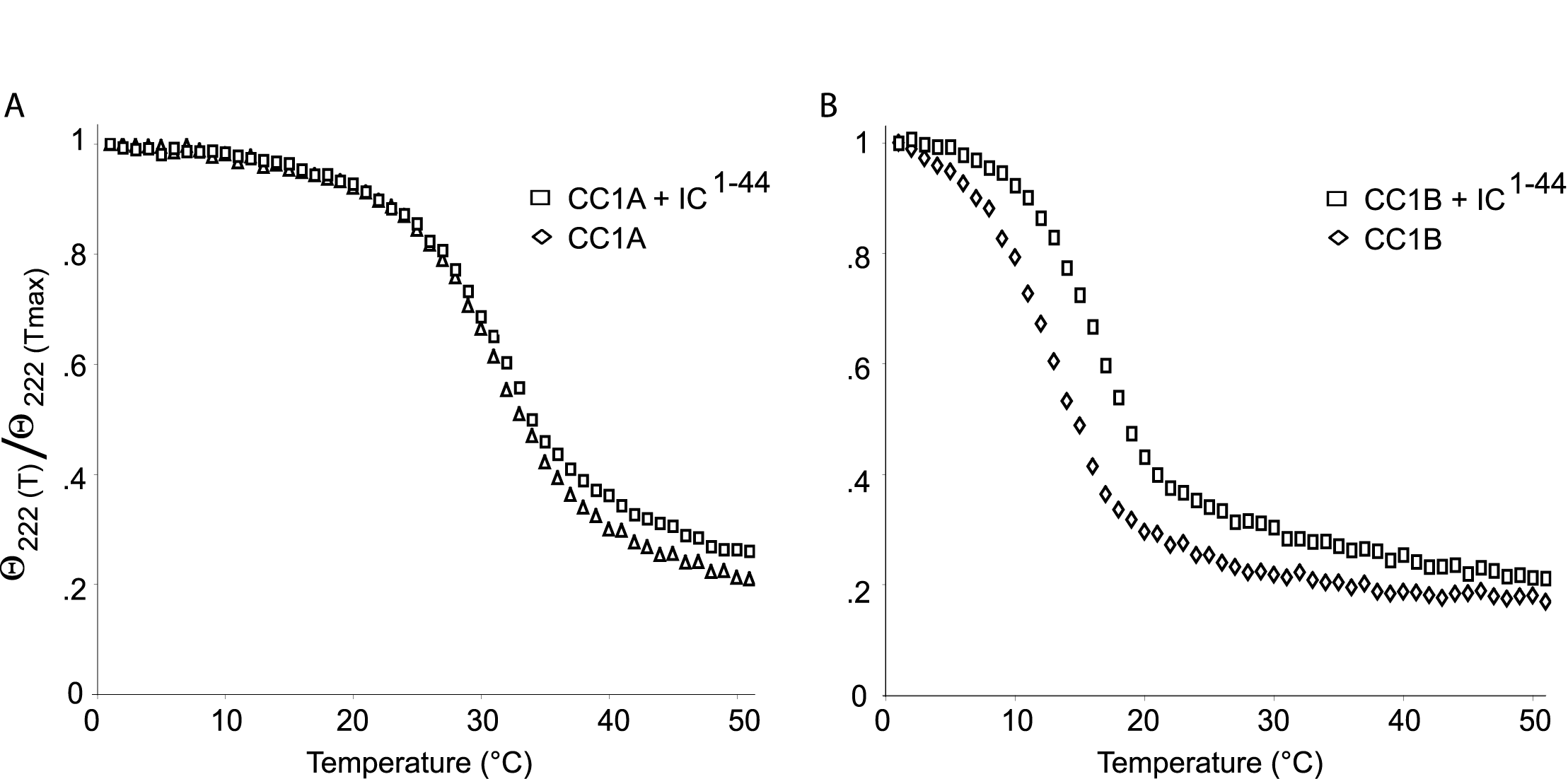

Supplement: Figure S6 — Thermal Denaturation of p150Glued fragments with IC1–44. (A) CC1A alone (diamonds) or incubated with an equimolar concentration of IC1–44 (squares). (B) CC1B alone (diamonds) or incubated with an equimolar concentration of IC1–44 (squares). (TIF) [file pone.0059453.s006.tif]

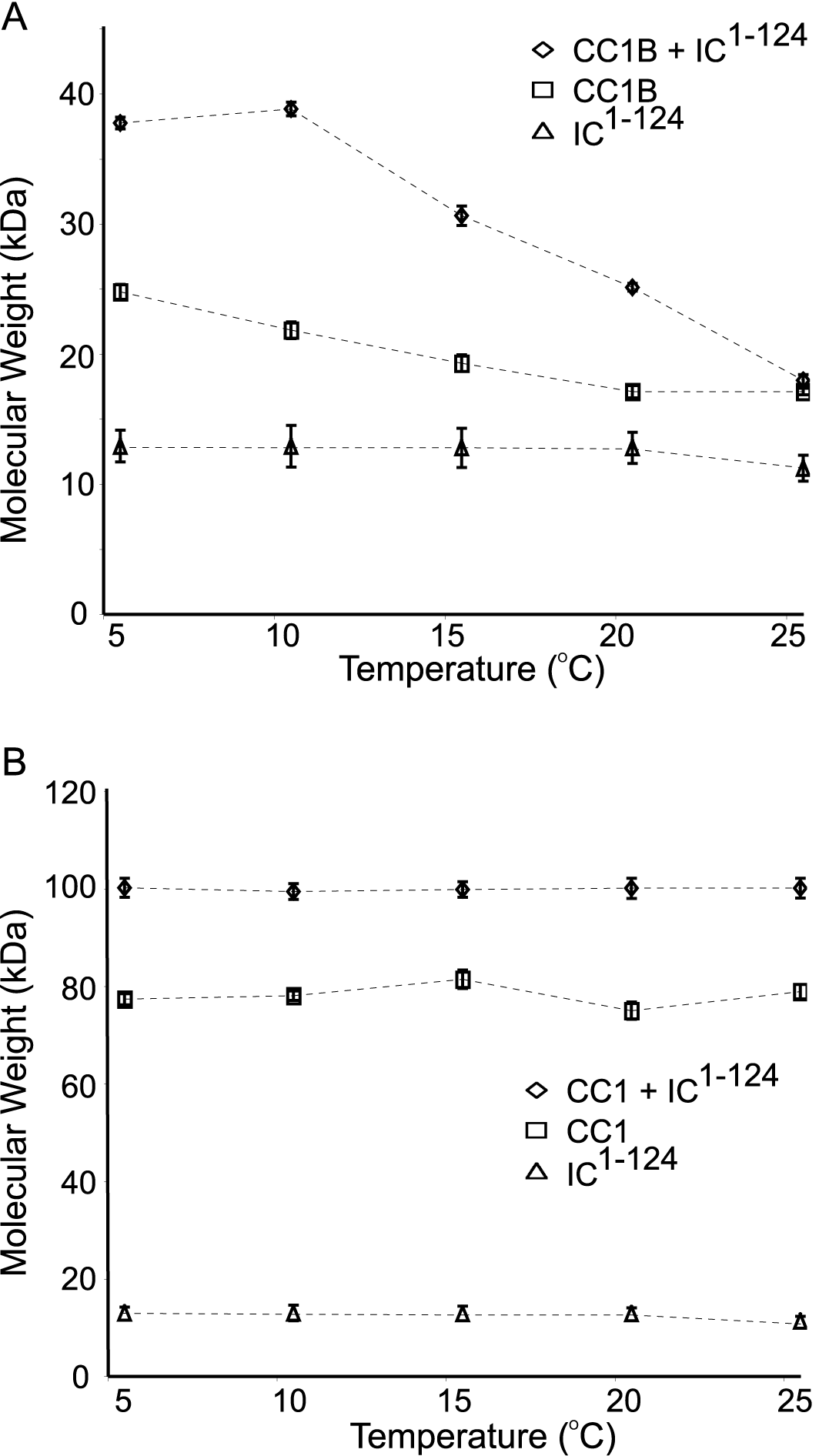

Supplement: Figure S7 — Dimerization and temperature dependence of IC-p150Glued interaction. (A/B) Oligomeric state and association with IC1–124 was examined as a function of temperature for both CC1B and CC1. A single speed of 25000 rpm was analyzed at 5, 10, 15, 20 and 25°C. The dimerization of CC1B and association with IC1–124 is temperature dependent (A), while no change in either dimerization or association is seen for CC1 (B). IC1–124 is monomeric at all temperatures. (TIF) [file pone.0059453.s007.tif]

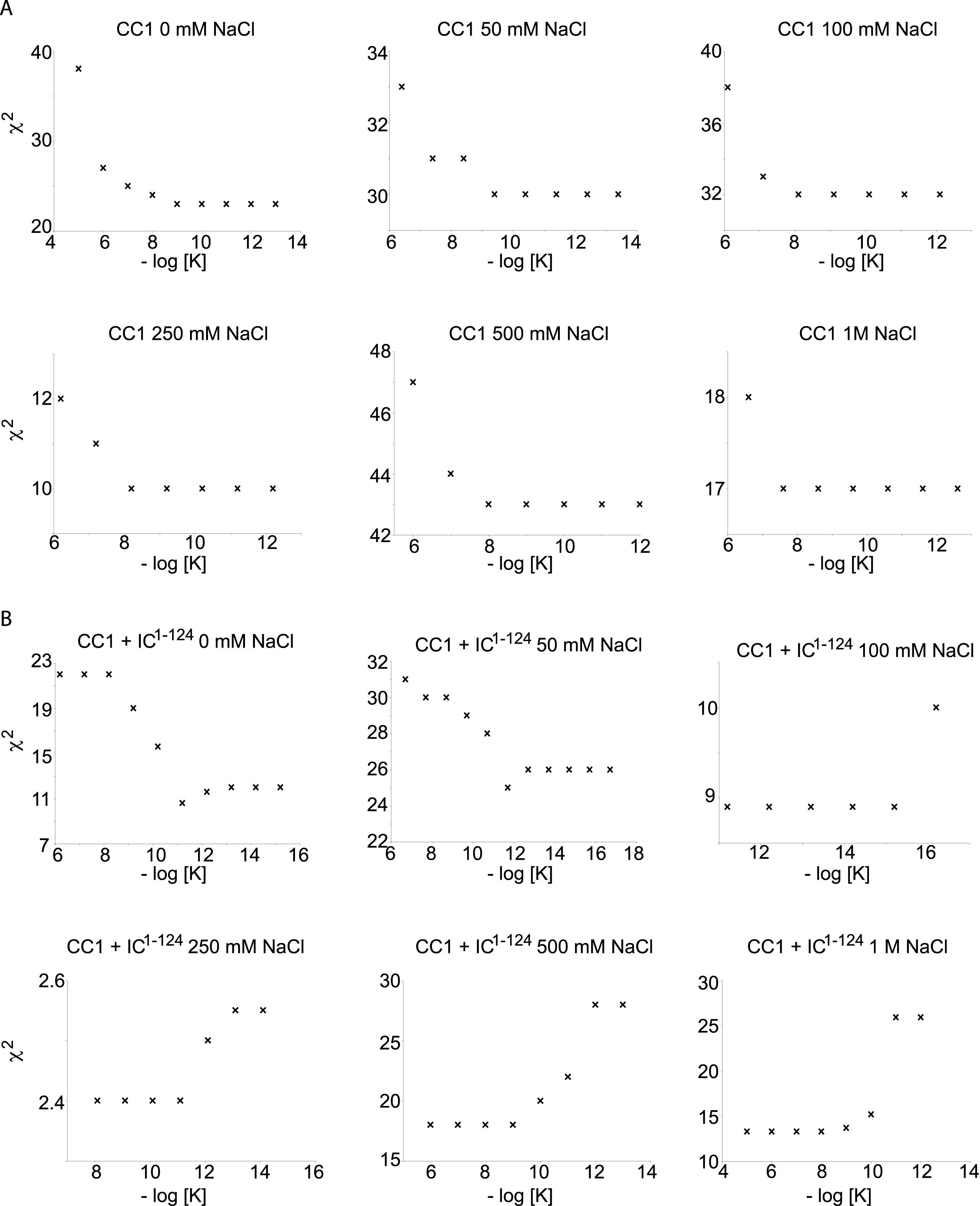

Supplement: Figure S8 — Chi square analysis of p150Glued oligomer (A) and IC-p150Glued complex (B) in salt dependence assays. (TIF) [file pone.0059453.s008.tif]

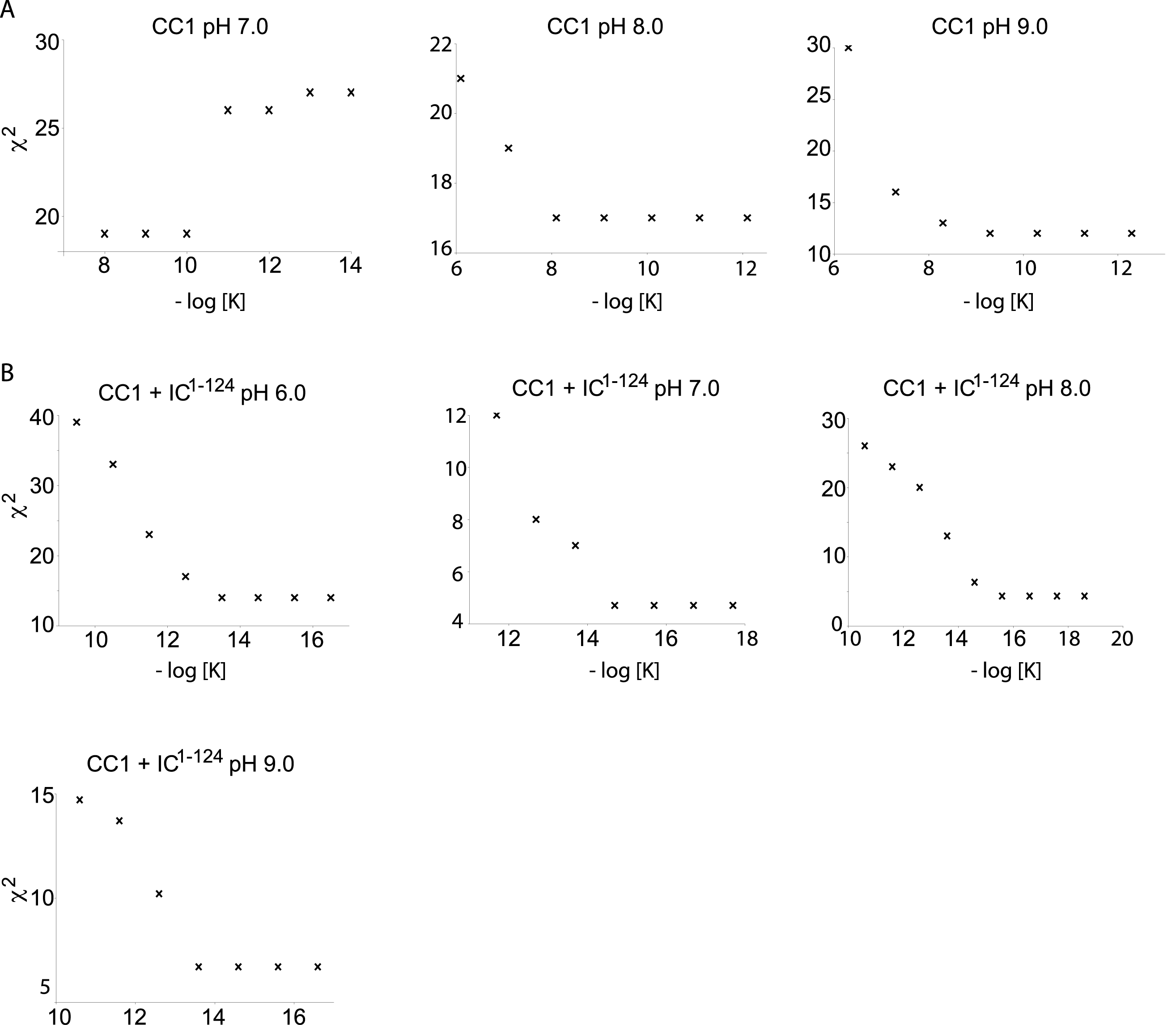

Supplement: Figure S9 — Chi square analysis of p150Glued oligomer (A) and IC-p150Glued complex (B) in pH dependence assays. (TIF) [file pone.0059453.s009.tif]
