# Supplementary figures and images for: Correction: Dynein and Dynactin Leverage Their Bivalent Character to Form a High-Affinity Interaction
Source: PLoS One. 2024 Jun 4;19(6):e0304916. doi: 10.1371/journal.pone.0304916 (PMC11149879; doi:10.1371/journal.pone.0304916)

## Slide 1
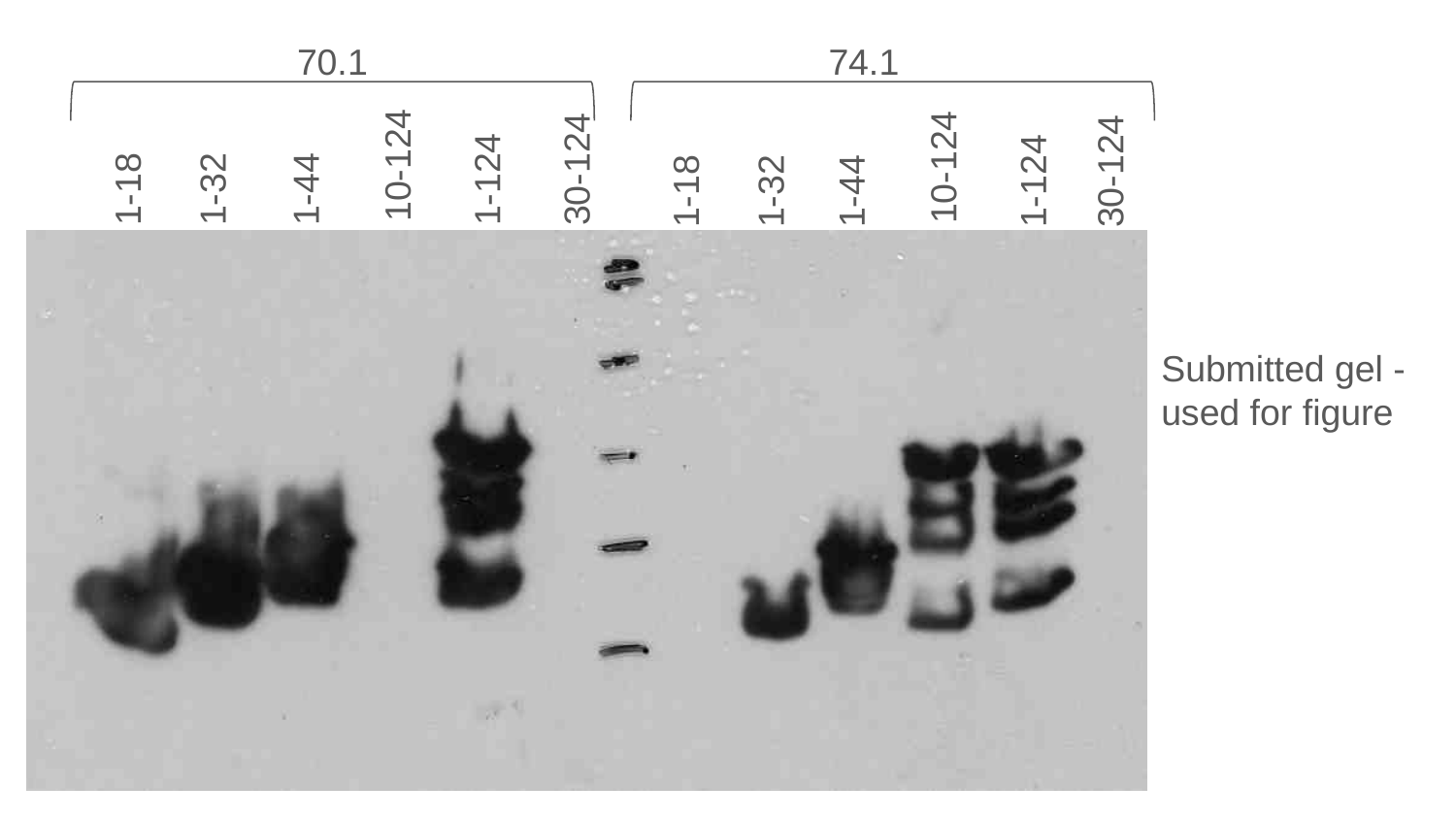

70.1
74.1
10-124
10-124
1-18
1-32
1-44
1-124
30-124
1-18
1-32
1-44
1-124
30-124
Submitted gel - used for figure

## Slide 2
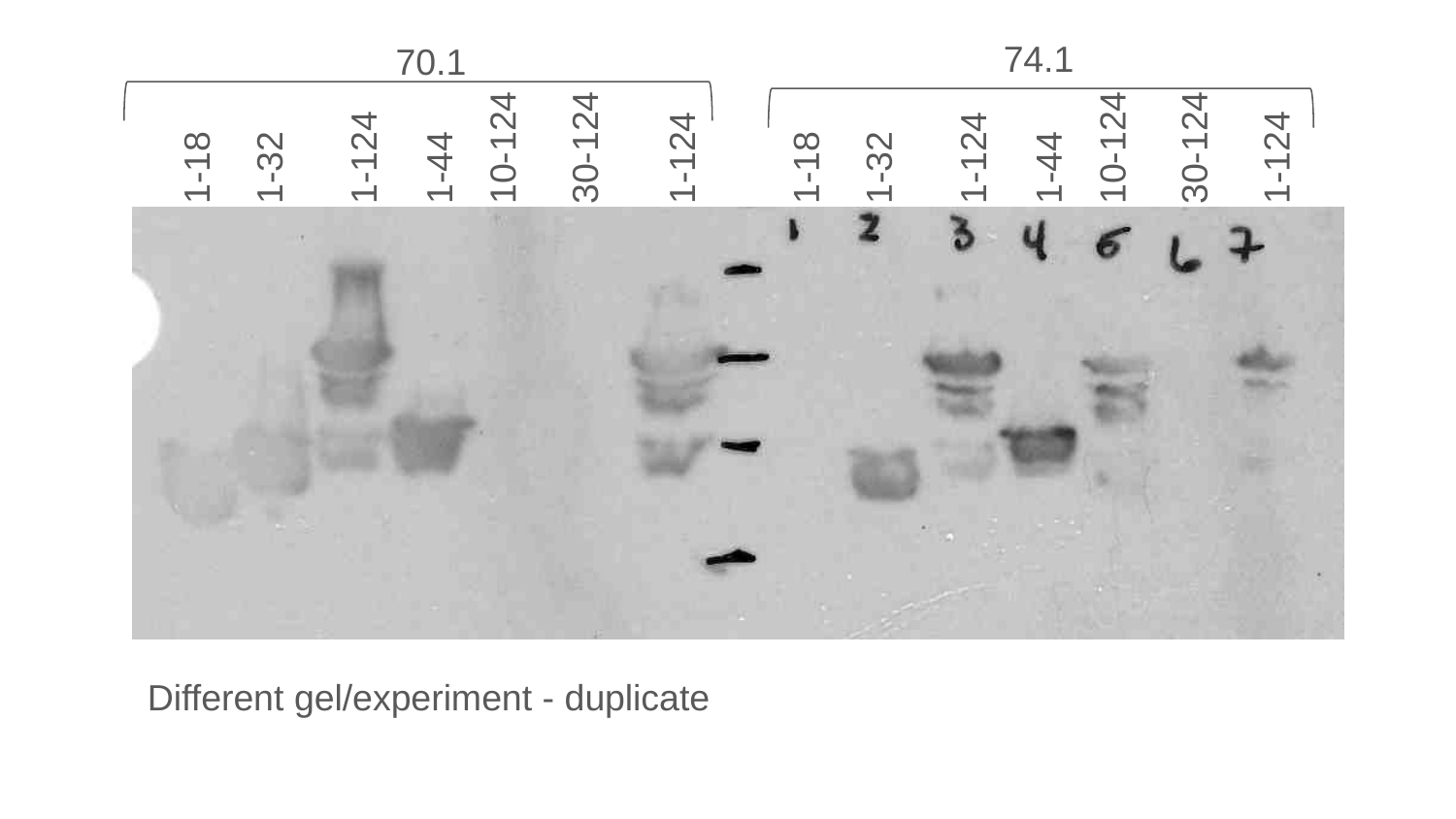

74.1
70.1
1-18
1-32
1-124
1-44
10-124
30-124
1-124
1-18
1-32
1-124
1-44
10-124
30-124
1-124
Different gel/experiment - duplicate

Supplement: S1 File — This file includes the original uncropped image used for Fig 2B on slide 1, and a repeat experiment, conducted at a later date, on slide 2. (PPTX) [file pone.0304916.s002.pptx]

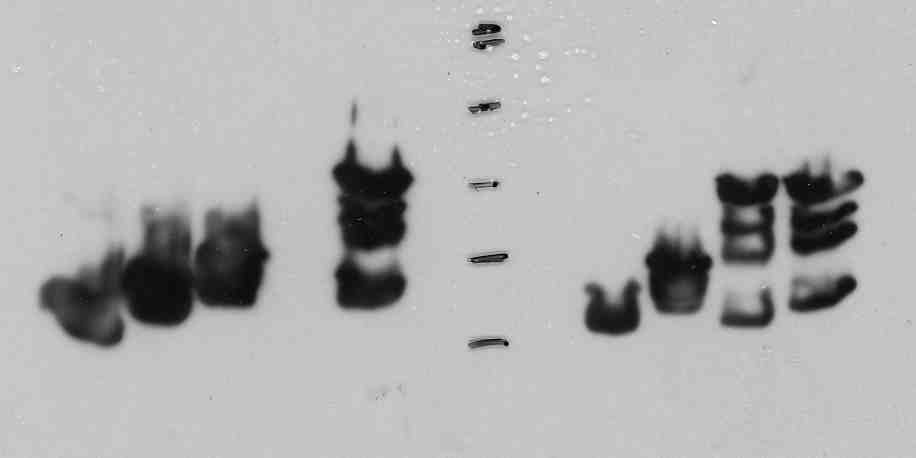

Supplement: S3 File — (JPG) [file pone.0304916.s004.jpg]

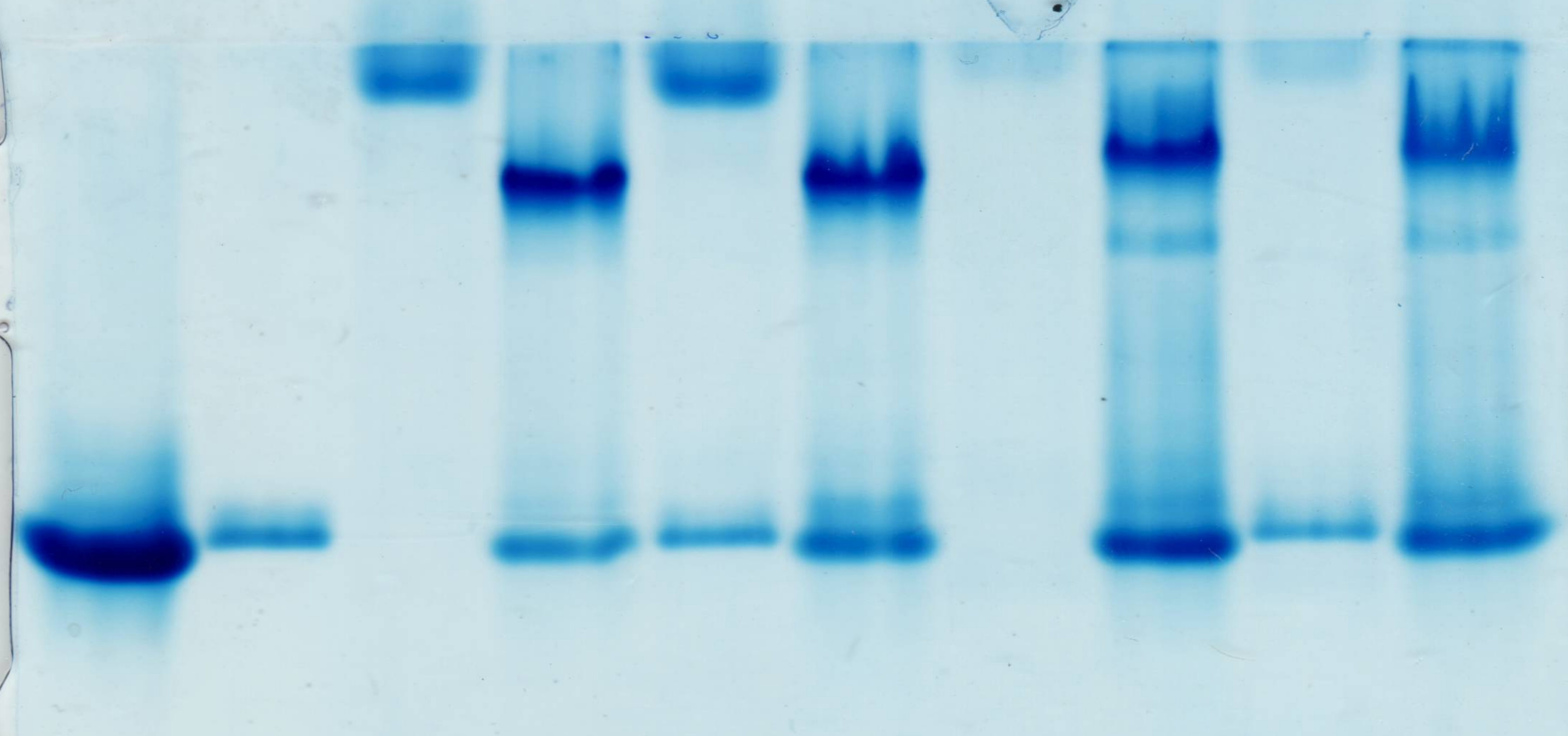

Supplement: S4 File — Original underlying image for gel presented in Fig 3B and S3A Fig. This file contains the original uncropped image for which lanes 3–6 correspond to lanes 7–10 of Fig 3B, and lanes 7–10 correspond to lanes 15–18 of S3A Fig. (PDF) [file pone.0304916.s005.pdf]

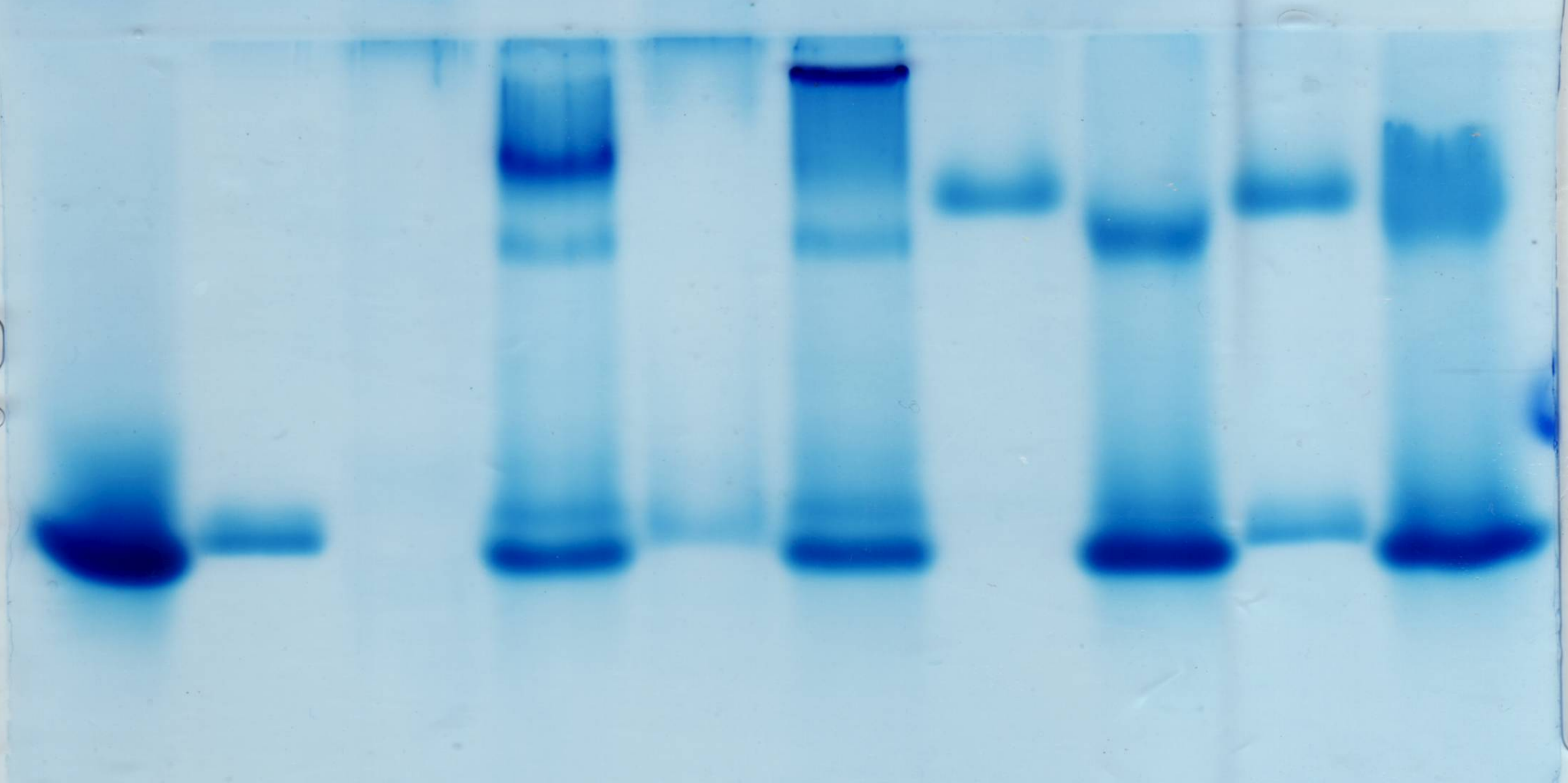

Supplement: S5 File — This file contains the original uncropped image for which lanes 1–6 correspond to lanes 1–6 of Fig 3B and lanes 7–10 correspond to lanes 3–6 of S3A Fig. (PDF) [file pone.0304916.s006.pdf]

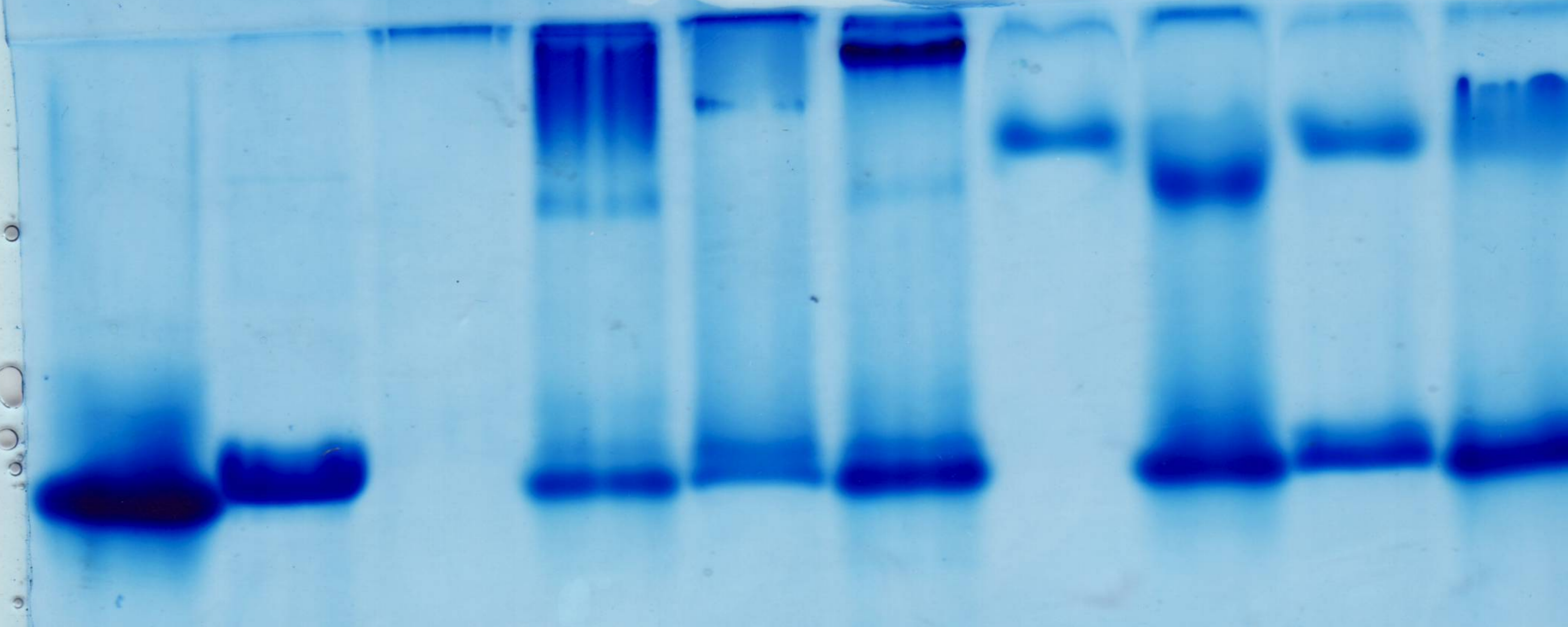

Supplement: S6 File — This file contains the original uncropped image for a repeat experiment conducted at an earlier date. Lanes 1–6 are a repeat experiment of lanes 1–6 in Fig 3B and lanes 7–10 are a repeat experiment of lanes 3–6 in S3A Fig. (PDF) [file pone.0304916.s007.pdf]

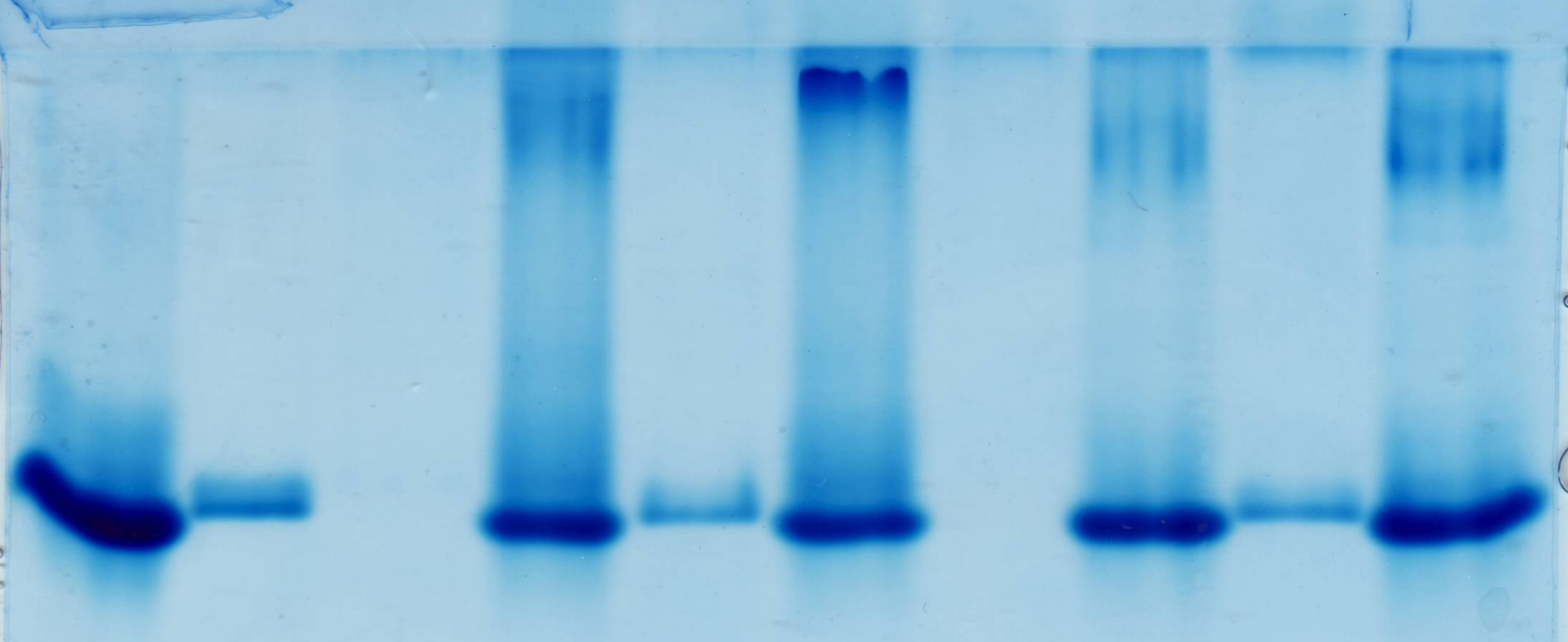

Supplement: S7 File — This file contains the original uncropped image for which lanes 3–10 correspond to lanes 7–14 of S3A Fig. (PDF) [file pone.0304916.s008.pdf]

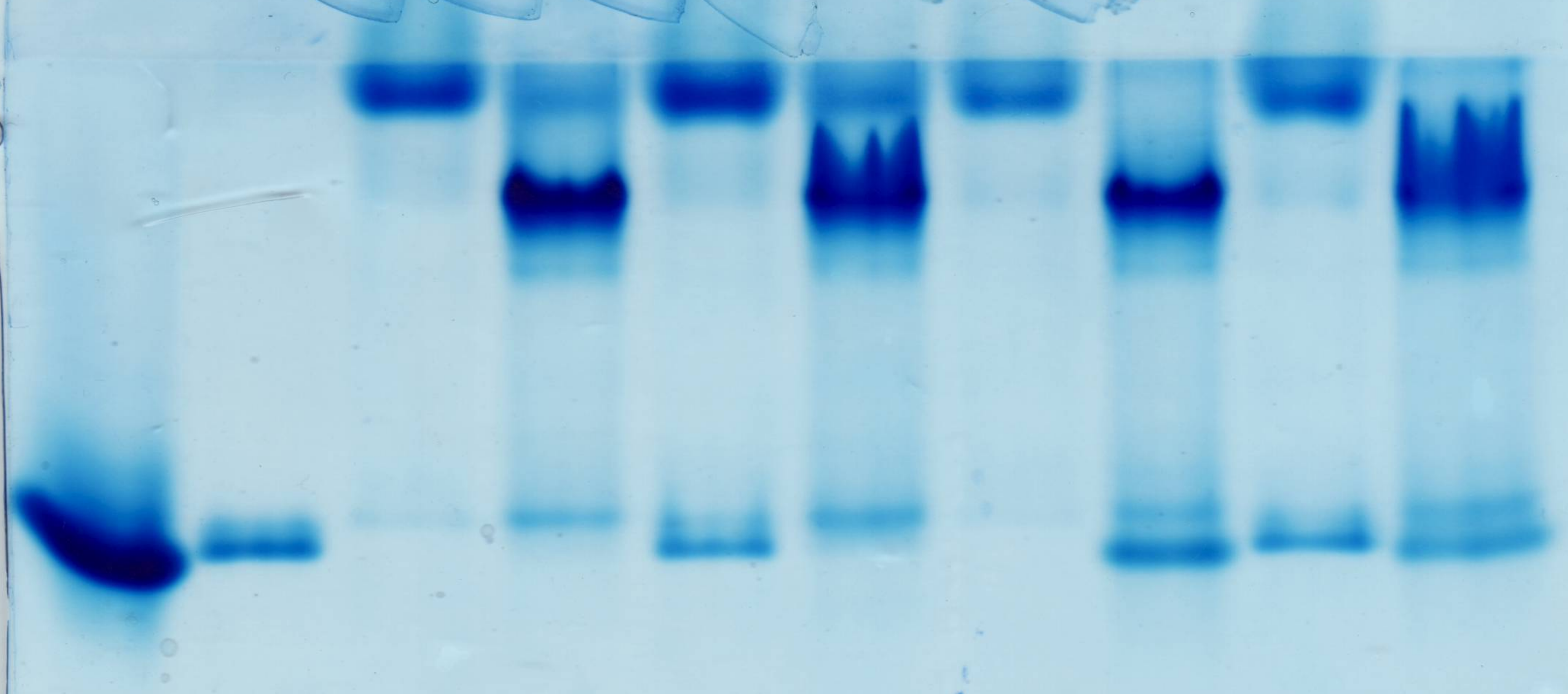

Supplement: S8 File — This file contains the original uncropped image for which lanes 1–10 correspond to lanes 1, 2 and 19–26 of S3A Fig. (PDF) [file pone.0304916.s009.pdf]
