# Supplementary material for: Correction: Dynein and Dynactin Leverage Their Bivalent Character to Form a High-Affinity Interaction
Source: PLoS One. 2024 Jun 4;19(6):e0304916. doi: 10.1371/journal.pone.0304916 (PMC11149879; doi:10.1371/journal.pone.0304916)
Supplement: S2 File — This file includes the two original uncropped images used to compose the updated Fig 3B on slide 1, and the four original uncropped images used to compose the updated S3A Fig on slides 2 and 3. (PPTX) [file pone.0304916.s003.pptx]

## Slide 1
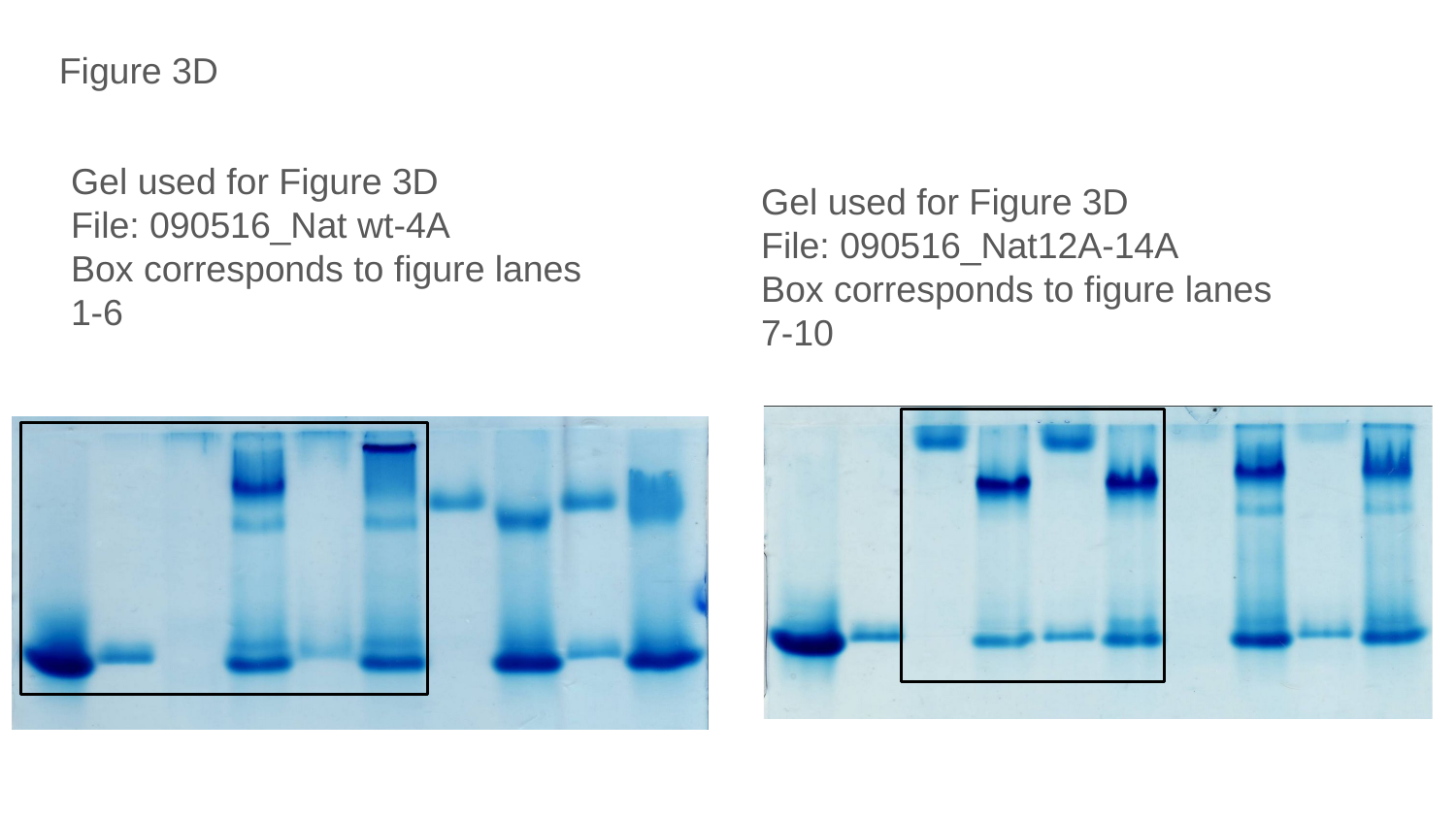

Figure 3D
Gel used for Figure 3D
File: 090516_Nat wt-4A
Box corresponds to figure lanes 1-6
Gel used for Figure 3D
File: 090516_Nat12A-14A
Box corresponds to figure lanes 7-10

## Slide 2
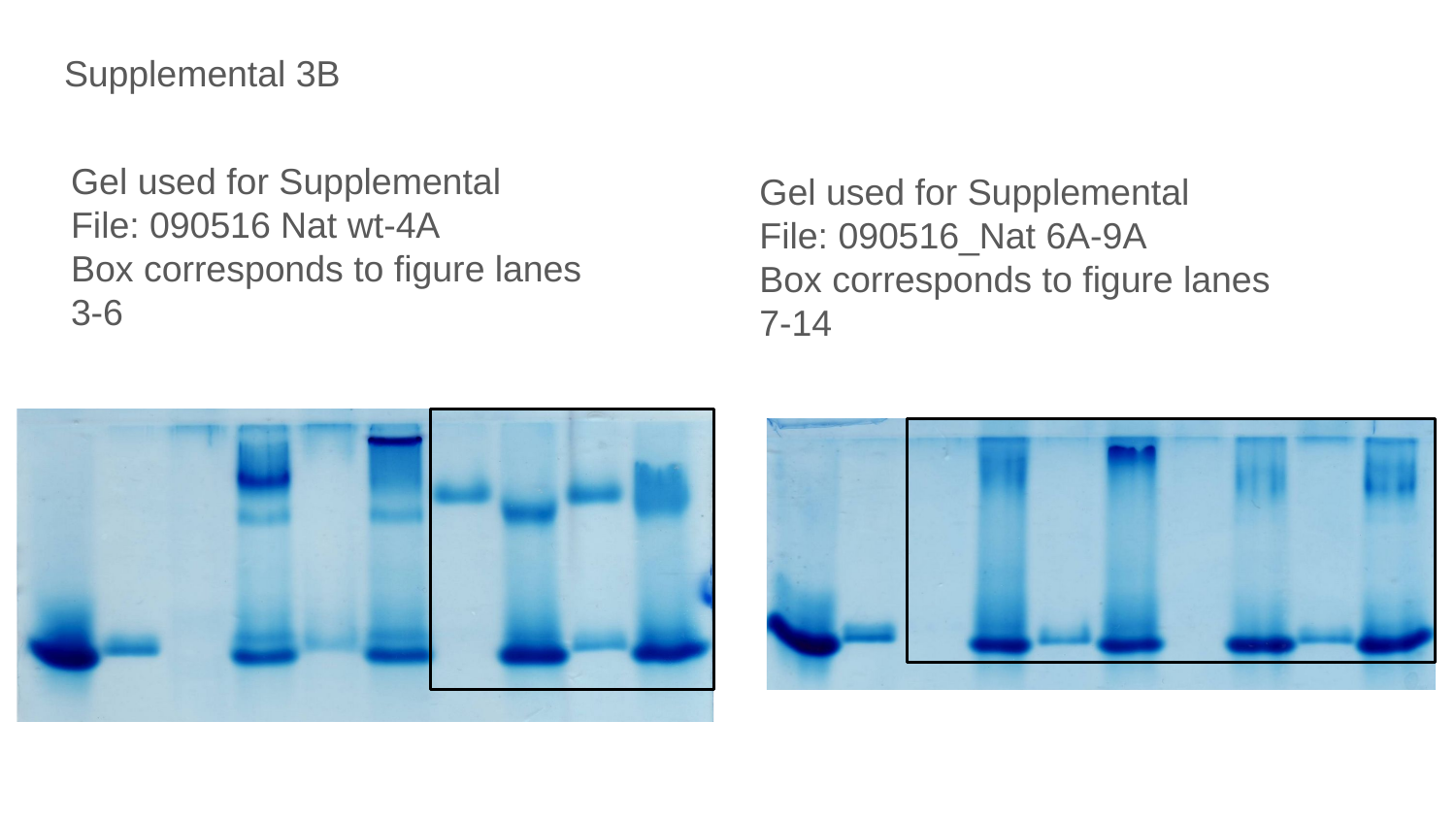

Supplemental 3B
Gel used for Supplemental
File: 090516 Nat wt-4A
Box corresponds to figure lanes 3-6
Gel used for Supplemental
File: 090516_Nat 6A-9A
Box corresponds to figure lanes 7-14

## Slide 3
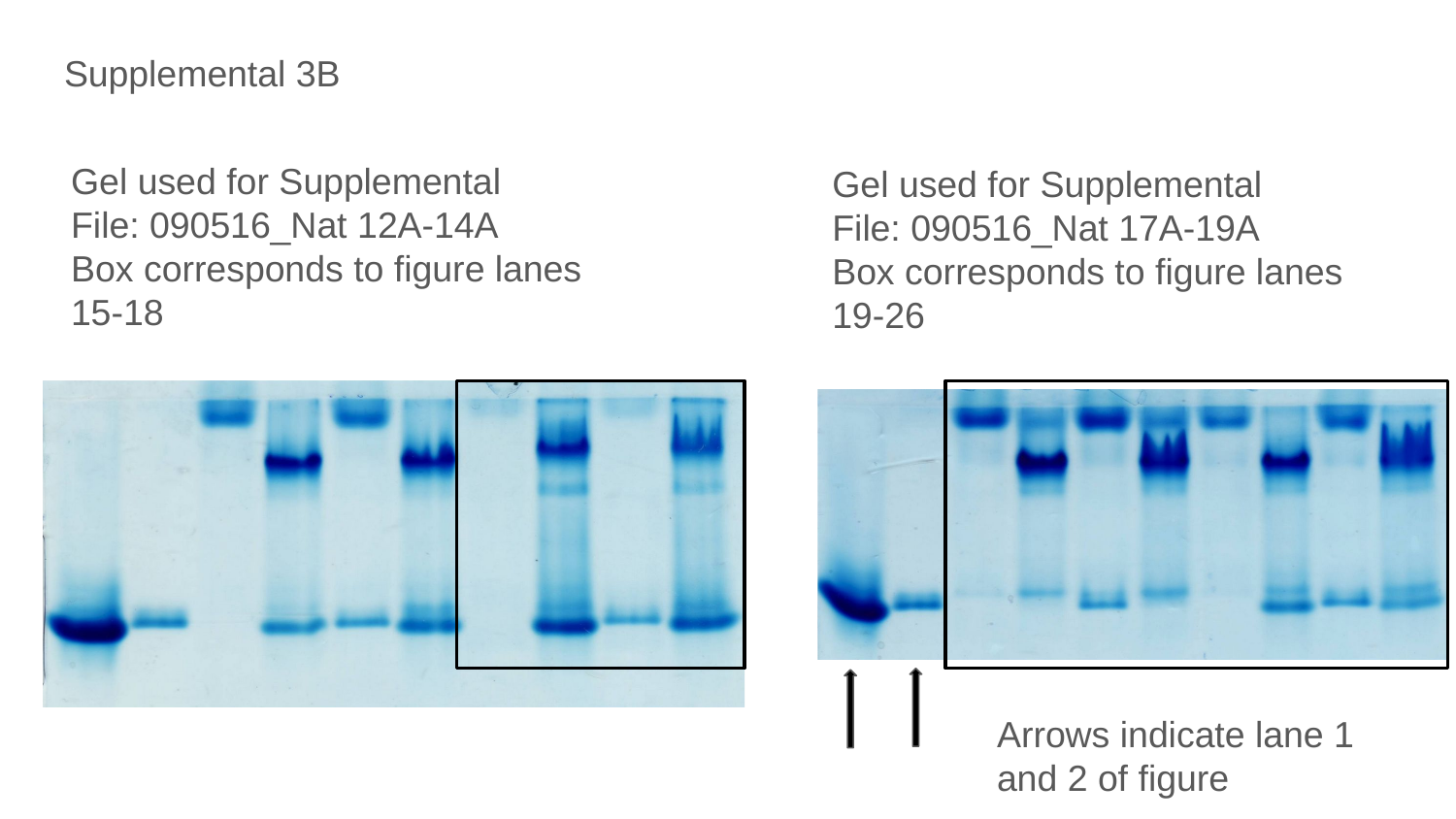

Supplemental 3B
Gel used for Supplemental
File: 090516_Nat 12A-14A
Box corresponds to figure lanes 15-18
Gel used for Supplemental
File: 090516_Nat 17A-19A
Box corresponds to figure lanes 19-26
Arrows indicate lane 1 and 2 of figure
