# Supplementary material for: Correction: Dynein and Dynactin Leverage Their Bivalent Character to Form a High-Affinity Interaction
Source: PLoS One. 2024 Jun 4;19(6):e0304916. doi: 10.1371/journal.pone.0304916 (PMC11149879; doi:10.1371/journal.pone.0304916)
Supplement: S3 Fig — (A) Native PAGE indicates that points mutants Pac11-L4A,K5A,Q6A, Pac11-Q6A,L7A,E8A, Pac11-E9A,K10A,R11A,Pac11-L17A,R18A, and Pac11-E19A,R20A,R21A abrogate Pac11-p150Glued CC1B binding. In the presence of LC8, Pac11-p150Glued CC1B binding is restored (indicated by an asterisk). Note only a slight change in migration is seen for the Pac11-p150Glued-LC8 complexes, however the CC1B band is absent or reduced indicating incorporation into the complex (arrow). Fig is composed of four separate native PAGE gels. (B) P-values were determined by t-test for mitotic spindle position assay. https://doi.org/10.1371/journal.pone.0059453.s003. (PDF) [file pone.0304916.s001.pdf]

A.

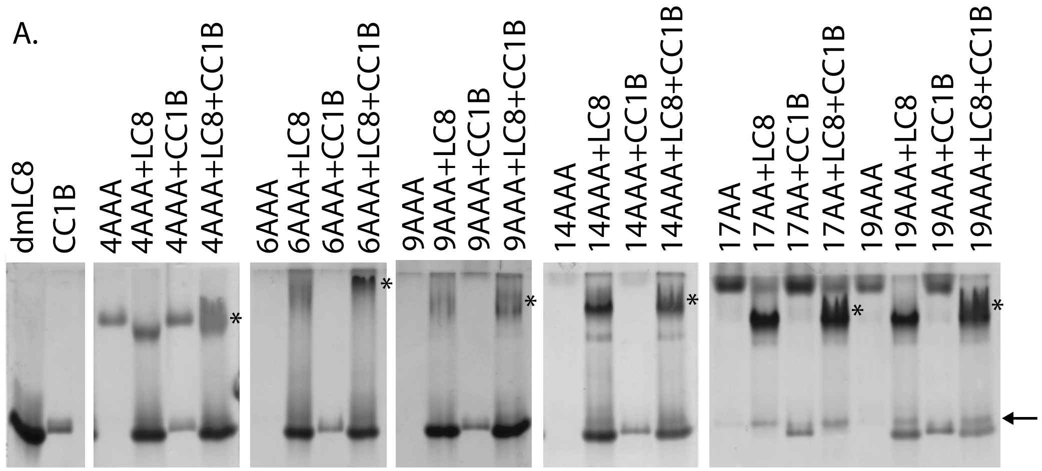

B.

	p-values determined by t-test								
	wt	nip100Δ	pac11Δ	4AAA	6AAA	9AAA	12AAA	17AA	19AAA
wt		<0.01	<0.01	0.09	<0.01	<0.01	<0.01	<0.01	<0.01
nip100Δ			0.39	<0.01	<0.01	<0.01	0.11	<0.01	<0.01
pac11Δ				<0.01	<0.01	<0.01	0.5	<0.01	<0.01
pac11-4AAA					<0.01	<0.01	<0.01	<0.01	0.62
pac11-6AAA						0.3	<0.01	0.76	<0.01
pac11-9AAA							<0.01	0.24	<0.01
pac11-12AAA								<0.01	<0.01
pac11-17AA									<0.01
pac11-19AAA									
